# Ultrawide-FOV full-color waveguides free of rainbow and fishbone artifacts via physics-constrained generative metagrating design

**DOI:** 10.1126/sciadv.aeh5234

**Published:** 2026-08-19

**Authors:** Mengguang Wang, Yong Li, Huihui Li, Fei Wu, Zeqing Yu, Huai Xia, Qiangbo Zhang, Changwei Zhang, Yiyang Liu, Huaze Xie, Chang Wang, Zhenrong Zheng

**Affiliations:** ^1^ College of Optical Science and Engineering, Zhejiang University, Hangzhou 310027, China.; ^2^ Beijing LLVision Technology Co., Ltd. Beijing 100176, China.; ^3^ Zhejiang Key Laboratory of Optoelectronic Information Technology, Hangzhou 310027, China.

## Abstract

Augmented reality (AR) displays are gaining prominence in consumer and industrial applications due to two-dimensional pupil-expanding waveguides, which achieve the essential dual requirements of compact form factors and large eyebox through innovative dual-axis beam replication technology. Nevertheless, these systems face an intrinsic trilemma compromising three critical parameters—efficiency, angular uniformity, and chromatic dispersion—with all three limitations becoming particularly pronounced at wide angles (>50°) across the visible spectrum, manifesting as fishbone artifacts (periodic non-uniformity patterns) and rainbow effects. To overcome these fundamental limitations, we present a physics-constrained generative adversarial network (PC-GAN) that systematically resolves the trilemma by incorporating two key physical principles: dilated pupil restraint ratio (DPRR) for uniformity control and periodic constraint theory (PCT) for dispersion management. This framework enables high-throughput discovery of metagrating designs while ensuring high-quality imaging performance in single-layer waveguides across the full visible spectrum and ultrawide field of view. Notably, the PC-GAN demonstrates its unique capability by autonomously discovering optimized snowflake-like metagrating (SLMG) geometries - fractal structures that provide breakthrough solutions for pupil expansion uniformity through their hierarchical light manipulation properties. Experimental realization of PC-GAN-optimized metagratings in disparate material systems (polymer and SiC) consistently delivered wide-angle, full-color operation in single-layer waveguides, while eliminating the characteristic fishbone artifacts and rainbow effects of conventional diffractive designs. This physics-constrained generative framework unlocks transformative potential for complex photonic systems beyond AR, enabling advanced designs ranging from ultrathin VR displays to multi-physics-optimized quantum optical devices.

## INTRODUCTION

Augmented reality (AR) represents a paradigm-shifting technological frontier that bridges digital information with our physical environment in real time. The field has witnessed remarkable progress since its emergence, driven by continuous innovations across optical engineering, novel materials, and display architectures. Among various implementation approaches, near-eye displays have emerged as the dominant platform for delivering immersive AR experiences, positioning themselves as potential successors to conventional computing interfaces (*1*). Current AR optical combiners face several fundamental limitations. Traditional solutions including birdbath configurations, freeform optical elements (*2*–*5*), retinal projection systems (*6*–*8*), and pin-mirror arrays (*9*, *10*) typically require intricate optical alignments. These architectures often incorporate multiple lens assemblies and voluminous projection optics, leading to compromised form factors. More critically, many systems struggle to achieve sufficient eyebox dimensions - the spatial region where virtual images remain perceptible to users. This parameter proves particularly vital given the natural variation in interpupillary distances among individuals and the constant ocular movements during environmental observation (*11*).

Waveguide-based architectures have emerged as a particularly promising solution for AR display combiners, owing to their ability to confine and manipulate light within thin, planar substrates through total internal reflection (TIR) (*12*, *13*). Among various waveguide implementations, two-dimensional (2D) pupil-expanding systems offer distinct advantages by enabling simultaneous beam replication along both horizontal and vertical axes within a single waveguide layer. This dual-axis expansion capability provides significant improvements in eyebox size and system compactness compared to conventional one-dimensional approaches (*12*, *14*). However, the development of high-performance 2D pupil-expanding waveguides faces a fundamental trilemma involving three critical performance parameters: diffraction efficiency, angular uniformity, and chromatic dispersion (*15*, *16*). The challenge stems from several intrinsic physical limitations: (i) Efficiency-uniformity trade-off–the cascaded diffraction in 2D pupil expansion creates progressively non-uniform energy distribution, manifesting as visible fishbone patterns (periodic luminance variations) that degrade image quality; (ii) Chromatic dispersion effects–the inherent wavelength dependence of diffractive optics induces angular separation of RGB components, generating pronounced rainbow effect; (iii) Multi-order interference–broadband operation (400–700 nm) produces uncontrolled higher-order diffraction that generates stray light, reducing contrast and creating ghost images (*17*–*19*). These interrelated challenges create a complex design space in which optimizing one parameter typically degrades the others. Current solutions, such as the multi-layer approach employed in commercial systems (e.g., 6 layers in Magic Leap 1 (*20*) or 3 layers in HoloLens), address the trilemma through physical separation of wavelength channels, but at the cost of increased thickness and fabrication complexity. The 2D single-layer alternative, while promising for its compact form factor, must overcome these fundamental physical constraints to achieve comparable performance.

Metasurfaces have emerged as a transformative platform for addressing the fundamental trilemma in AR waveguide displays, offering unprecedented control over light manipulation through subwavelength nanostructures (*21*–*26*). These artificial optical elements enable precise engineering of phase, amplitude, and polarization via various physical mechanisms, demonstrating remarkable potential for display applications (*27*–*32*). While recent advances in metasurface-based waveguide couplers have shown promising improvements in angular bandwidth, diffraction efficiency and field-of-view, the simultaneous optimization of these competing parameters across the full visible spectrum and wide angular range remains an extraordinary challenge (*33*–*39*). The complex interdependencies between material dispersion, nanostructure geometry and multi-wavelength interference create a vast, non-linear design space that conventional optimization methods struggle to navigate effectively (*23*, *40*–*47*). Although multilayer architectures provide additional degrees of freedom for dispersion control, they introduce significant fabrication complexities and performance trade-offs (*48*). This critical limitation motivates our development of a generative adversarial network enabling the realization of high-performance, single-layer metasurface couplers capable of overcoming the longstanding efficiency-uniformity-dispersion trilemma.

**Fig. 1.**
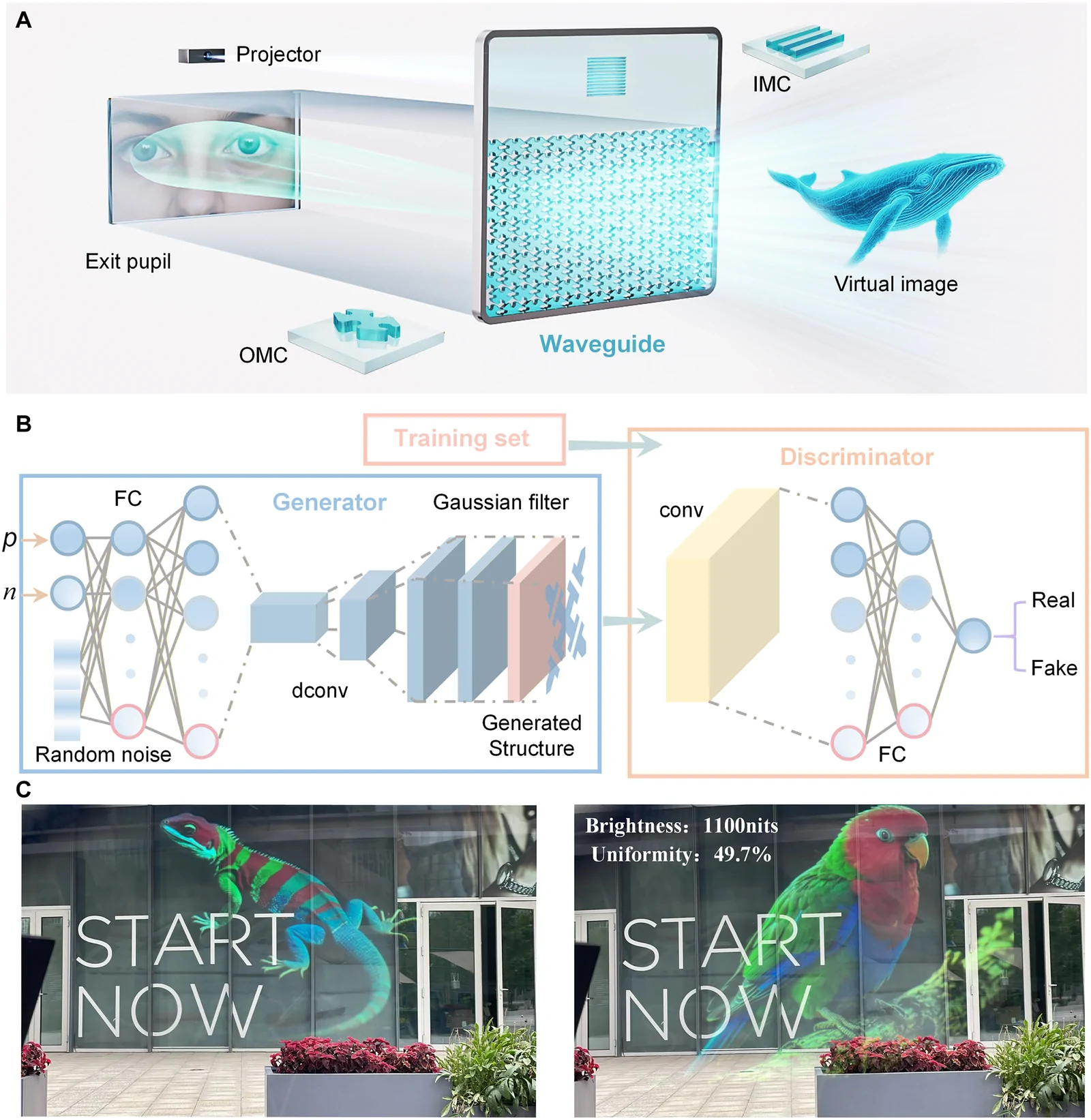
Schematic illustration of metasurface waveguide AR display system and schematic diagram of PC-GAN. (**A**) The AR optical system employs a novel waveguide architecture incorporating dual-function metasurface couplers within a high-index transparent substrate. An input metasurface coupler (IMC) efficiently injects projected imagery into the waveguide, where light undergoes two-dimensional expansion through successive total internal reflection events. Unlike conventional designs, this configuration enables simultaneous beam replication along both horizontal and vertical axes while preserving ambient light transmission. The expanded exit pupil is then redirected toward the viewer’s eye by the OMC, maintaining optical see-through capability. This dual-axis expansion mechanism provides significant advantages in eyebox size and optical efficiency compared to traditional one-dimensional waveguide implementations, while the single-layer construction ensures minimal thickness and weight. The system’s unique light management combines diffractive beam steering with refractive propagation characteristics, achieving wide field-of-view projection without compromising see-through clarity or form factor. (**B**) Schematic diagram of PC-GAN. The PC-GAN architecture for generating metagratings features a generator network with two fully connected (FC) layers and four deconvolution (dconv) layers, culminating in Gaussian smoothing, while the discriminator employs a single convolutional layer followed by two fully connected layers. Following the training phase, the generator outputs intricate topological patterns optimized for specific period (*p*) and refractive index (*n*) requirements. (**C**) Images captured of outdoor daytime scenes in the augmented reality mode.

In this study, we present our physics-constrained generative adversarial network (PC-GAN) as a comprehensive solution that fundamentally transforms metasurface design for AR waveguide applications (Fig. 1). The proposed framework successfully integrates nanophotonic physics with deep learning to generate manufacturable metasurface architectures that simultaneously achieve reasonable multi-level diffraction efficiency, exceptional angular uniformity, and minimal chromatic dispersion in a single-layer configuration. Experimental validation using both polymer (*n* = 1.7) and silicon carbide (SiC, *n* = 2.6) platforms demonstrates the universality of our approach, with fabricated prototypes exhibiting complete elimination of fishbone artifacts and rainbow effects while maintaining compact waveguide thickness–the SiC-based device achieves a remarkable 75° field of view (FOV) with a brightness of 1100 nits, an average spatial frequency of 15 cycles per mm maintained at modulation transfer function (MTF) >0.6, and luminance uniformity of 49.7% across the full visible spectrum (the uniformity of the conventional two-dimensional waveguide is less than 20%). These results establish PC-GAN as a powerful paradigm for overcoming the longstanding trilemma in AR display technology, while the physics-constrained architecture ensures reliable performance across the full visible spectrum without requiring complex multilayer stacks. The success of this approach opens possibilities for applying constrained generative design to other challenging problems in nanophotonics and optical engineering.

## RESULTS

### Efficiency analysis

**Fig. 2.**
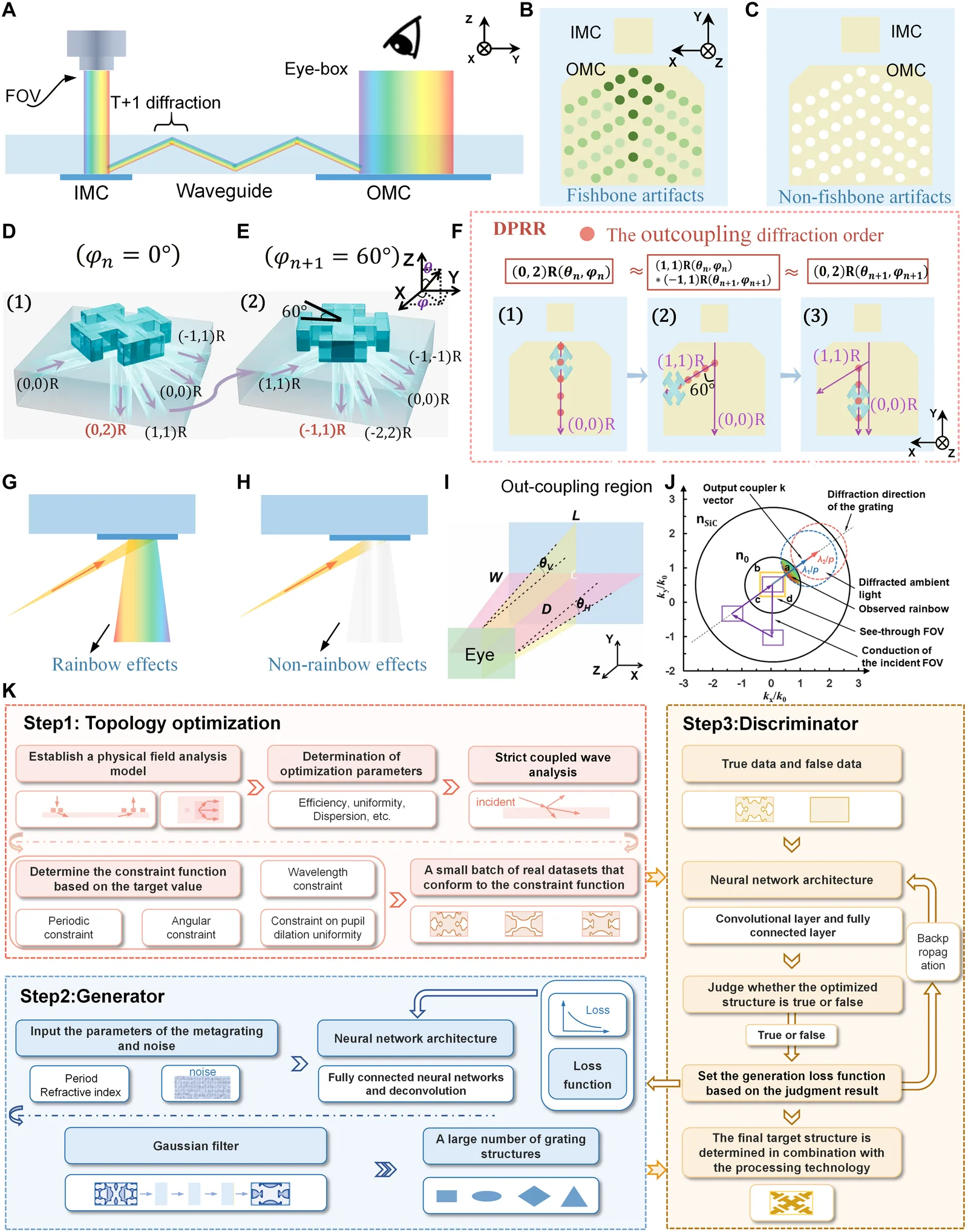
Physical constraints of metasurface waveguide and the architecture of PC-GAN. (**A**) Schematic illustration of the optical path comprising components such as the micro-display and waveguide. (**B**) Schematic diagram of fishbone artifacts generated by traditional diffracted optical waveguides. The brightness of the green light spot represents the size of the output diffraction order. (**C**) The ideal exit-pupil uniformity required for high-quality AR displays. The brightness of the white light spot represents the size of the output diffraction order. (**D**) The diffraction situation of the metasurface grating when the $\varphi_{n}$ azimuth angle is 0°. (**E**) The diffraction situation of the metasurface grating when the $\varphi_{n+1}$ azimuth angle is 60°. (−1, 1) R represents the diffraction order that enters the human eye. The purple arrow indicates the direction of the light path. (**F**) The planar optical path diagram for two-dimensional expansion of the light beam using optical waveguides. The red spots represent the diffraction orders that are coupled into the human eye. (**G** and **H**) Schematic diagrams of the optical paths for the generation of reflected rainbow patterns and non-reflected rainbow patterns by ambient light. (**I**) illustrates the geometric configuration of the waveguide exit pupil (output coupler) region, specifying the horizontal (θ_H_) and vertical (θ_V_) viewing angles relative to the observer’s position, where *D* denotes the eye relief distance to the output coupling region. The corresponding *k*-space representation in (**J**) depicts the light propagation characteristics, with the inner and outer circles representing the normalized wavevectors in air and SiC (*n* = 2.6) respectively. Wavelength-dependent dispersion is indicated by the shifted blue (450 nm) and red (650 nm) annular regions, offset by λ_i_/*p* along the grating’s diffraction axis (where λ_i_ is the incident wavelength and *p* the grating period). (**K**) The process schematic diagram of PC-GAN.

Figure 2A presents the cross-sectional schematic of the waveguide architecture, illustrating the light propagation mechanism between the input and output metasurface couplers. To achieve full-color performance across an extended field-of-view, our PC-GAN framework implements simultaneous optimization of 27 key parameters in the output metasurface coupler (OMC) design–specifically addressing the three diffraction orders (0, 2), (1, 1) and (−1, 1) for RGB wavelengths (460 nm, 530 nm, 620 nm) at three distinct incident angles (40°, 60°, 80°). This comprehensive multi-parameter constraint scheme effectively resolves the inherent multi-order diffraction efficiency challenge while maintaining optimal light management throughout the visible spectrum. The coordinated optimization of these interdependent variables enables precise control over both chromatic dispersion and angular response, ensuring uniform image quality across the entire operational bandwidth.

### Uniformity analysis

Figure 2B illustrates the characteristic fishbone artifacts (periodic luminance non-uniformity) commonly observed in conventional diffractive waveguides. These artifacts arise from the interference of cascaded diffraction processes during two-dimensional pupil expansion, typically reducing the MTF below 0.2 and causing perceptible luminance variations exceeding 50%. In contrast, Fig. 2C depicts the ideal exit-pupil uniformity required for high-quality AR displays, where the intensity distribution should maintain a small variation across the entire eyebox.

To address this fundamental challenge, we analyzed the diffraction physics governing multi-stage pupil expansion and identified that, in addition to efficiency decay across diffraction orders, variations in outcoupling diffraction orders at two specific azimuthal angles also contribute significantly to non-uniformity: (i) the first case involves a grating azimuth of 0° for longitudinal pupil expansion, as shown in Fig. 2D, where the outcoupling diffraction orders are (0, 2) R, and (ii) the second case corresponds to lateral pupil expansion at a grating azimuth of 60° (Fig. 2E), with outcoupling orders of (−1, 1) R. Notably, the input orders for the latter originate from the output orders (1, 1) R of the longitudinal expansion case (the detailed process of the pupil dilation in the two-dimensional optical waveguide can be found in note S1). These two pupil expansion processes serve as the primary expansion modes within the waveguide, operating continuously and repetitively. Moreover, due to the inherent symmetry of the grating, the behavior observed at an azimuth angle of −60° remains consistent with that at 60°. To enhance uniformity by balancing the efficiency among outcoupling diffraction orders, we further derived a novel dilated pupil restraint ratio (DPRR)

$$\text{DPRR}=\left(1,1\right)R\left(\theta_{n},\varphi_{n}\right)*\left(-1,1\right)R\left(\theta_{n+1},\varphi_{n+1}\right)/\left(0,2\right)R\left(\theta_{n},\varphi_{n}\right)$$ (1)

where $\theta_{n}$ and $\varphi_{n}$ denote the polar and azimuthal angles of light incident on the OMC during the *n*th interaction, while $\theta_{n+1}$ and $\varphi_{n+1}$ represent the corresponding angles during the (*n* + 1) th interaction. In this framework, $\left(0,2\right)R\left(\theta_{n},\varphi_{n}\right)$ corresponds to the diffraction order coupling light directly to the eye during the *n*th OMC interaction, $\left(1,1\right)R\left(\theta_{n},\varphi_{n}\right)$ represents the orders propagating laterally for pupil expansion, and $\left(-1,1\right)R\left(\theta_{n+1},\varphi_{n+1}\right)$ describes the diffraction order coupling light directly to the eye during the (*n* + 1) th interaction. This design is implemented because the key pupil expansion modes in 2D waveguides can be summarized into two types (Fig. 2F): (i) direct out-coupling $\left(0,2\right)R\left(\theta_{n},\varphi_{n}\right)$, and (ii) secondary expansion coupling $\left(1,1\right)R\left(\theta_{n},\varphi_{n}\right)*\left(-1,1\right)R\left(\theta_{n+1},\varphi_{n+1}\right)$. By treating these two modes equivalently, with DPRR approximately equal to 1, we ensured the uniformity of pupil dilation (note S2). This selective optimization focuses exclusively on these dominant modes–which are fundamental to two-dimensional waveguide pupil expansion–while intentionally excluding less compatible higher-order or asymmetric couplings that could compromise uniformity. The resulting configuration guarantees uniform brightness across the expanded pupil without introducing parasitic artifacts, achieving optimal performance through physically constrained mode selection rather than exhaustive consideration of all possible diffraction orders.

### Dispersion analysis

In AR waveguide-based optical systems, chromatic dispersion-induced rainbow effect presents a fundamental limitation that significantly degrades visual quality and user experience. This phenomenon primarily originates from wavelength-dependent diffraction of ambient light at the output coupler interface when external illumination directly interacts with the grating region, as shown in Fig. 2, G and H. To mitigate this challenge and streamline the diffractive waveguide design methodology, we introduce a periodic constraint theory (PCT) framework that enables more intuitive and computationally efficient optimization of grating parameters. Notably, the light propagation mechanism in waveguide output couplers exhibits analogous behavior to input couplers, with only higher-order diffracted modes possessing sufficient momentum to couple out of the waveguide and form visible images at the eye position.

Figure 2I illustrates the geometric configuration of the waveguide’s exit pupil region, characterized by length (*L*), width (*W*), eye relief distance (*D*), horizontal field-of-view (θ_H_), and vertical field-of-view (*θ*_V_). The corresponding *k*-space representation, shown in Fig. 2J, transforms these real-space boundaries into a single rectangular region in k-space, defined by four vertices a–d with coordinates $\left(L/\sqrt{4D^{2}+L^{2}},W/\sqrt{4D^{2}+W^{2}}\right)$, $\left(-L/\sqrt{4D^{2}+L^{2}},W/\right.\left.\sqrt{4D^{2}+W^{2}}\right)$, $\left(-L/\sqrt{4D^{2}+L^{2}},-W/\sqrt{4D^{2}+W^{2}}\right)$, and $\left(L/\sqrt{4D^{2}+L^{2}},\right.\left.-W/\sqrt{4D^{2}+W^{2}}\right)$ respectively. When analyzing chromatic dispersion effects, ambient light with wavelength λ incident on the output coupler at oblique angles can be modeled as a unit circle in *k*-space displaced by λ/*p* along the grating vector direction, where *p* represents the output grating period. The condition for rainbow effect generation occurs when this displaced circle intersects with the see-through FOV region. To prevent such chromatic artifacts across the entire operational spectrum, the horizontal grating period px and the vertical grating period py must satisfy

$$\frac{\lambda_{\text{Red}}}{\text{sin}\left(\varphi_{t}/2\right)\;\left(n-\text{sin}\theta_{\text{in}}\right)}<\mathrm{px}\leq \frac{\lambda_{\text{Blue}}}{\text{sin}\left(\varphi_{t}/2\right)\;\left(1+\text{sin}\theta_{\text{max}}\right)}$$ (2)

$$\mathrm{py}=\mathrm{px}\cdot \text{tan}\left(\varphi_{t}/2\right)$$ (3)

In the formula, the incident angle is $\theta_{\text{in}}$, while λ_Red_ = 650 nm and λ_Blue_ = 450 nm denote the characteristic wavelengths for red and blue light components respectively, and $\varphi_{t}$ in our design is 60°. The parameter $\theta_{\text{max}}$ defines the maximum angular deviation between the incident light propagation vector and the waveguide substrate normal, effectively representing the human eye’s maximum viewing angle within the system’s operational range. This dual requirement ensures complete suppression of wavelength-dependent diffraction while maintaining optimal waveguide performance. The geometric transformation between real-space and *k*-space parameters provides a rigorous framework for analyzing and mitigating rainbow effects in diffractive waveguide systems. The detailed derivation process and simulation results are presented in notes S3 and S4.

### The framework of PC-GAN

The PC-GAN architecture integrates fundamental optical principles with deep learning through three synergistic components (Fig. 2K): a topology optimization module that generates physically-valid metagrating configurations by solving Maxwell’s equations with constrained boundary conditions (note S5), a generator network that incorporates differentiable physical layers to enforce wave optics constraints during the synthesis process, and a hybrid discriminator that simultaneously evaluates structural authenticity and physical feasibility. The generator’s unique physics-embedded architecture transforms random noise into viable designs by maintaining strict adherence to the DPRR and PCT throughout the generation process, while the discriminator’s dual-path structure ensures both geometric plausibility and optical performance requirements are met. This framework fundamentally differs from conventional GANs by embedding the complete physical forward model–including wave propagation, diffraction efficiency, and chromatic dispersion relationships–directly into the network’s loss landscape, enabling the discovery of complex, non-intuitive metasurface geometries that inherently satisfy multiple competing optical constraints. The adversarial training process naturally explores the Pareto frontier of the multi-objective optimization space, balancing efficiency, uniformity, and dispersion characteristics through physically-grounded competition between generator and discriminator, while the integrated topology optimization prior provides strong physical guidance to accelerate convergence toward feasible solutions. By constructing this tight coupling between deep learning and first-principles physics, PC-GAN establishes a paradigm for nanophotonic inverse design that intrinsically bridges the gap between computational exploration and physical realizability.

### The performance of PC-GAN

**Fig. 3.**
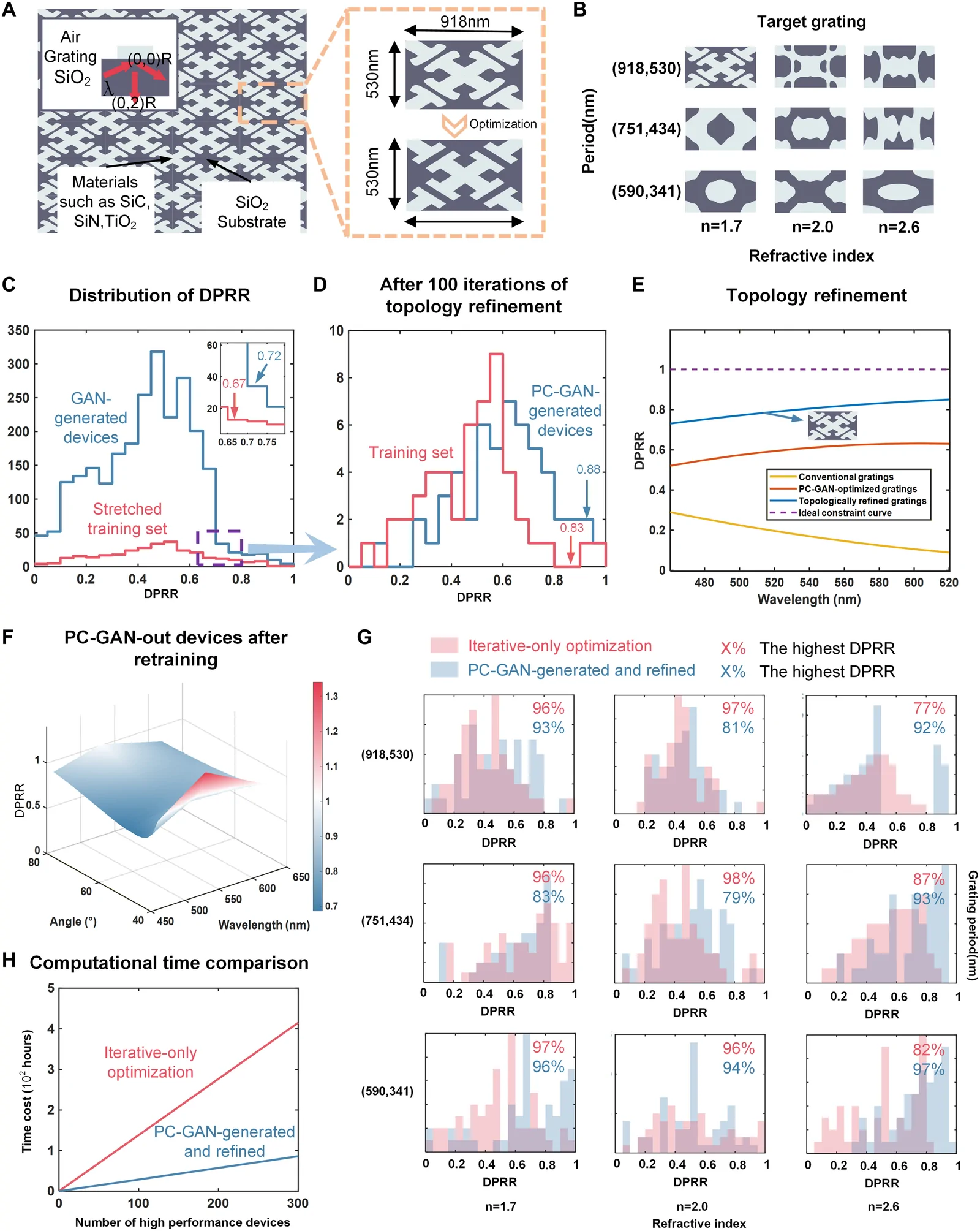
The performance of PC-GAN. (**A**) A top-view image of a representative PC-GAN optimized SLMG structure designed for efficient light coupling into specified diffraction orders and achieve uniform pupil dilation. The nanostructure comprises an 80 nm-thick materials (SiN, TiO_2_) thin film patterned on a glass substrate, with critical dimensions optimized for visible spectrum operation (450–650 nm). For the generative modeling framework, each metagrating unit cell was represented as a 1000 × 800 pixel binary mask, capturing the detailed nanoscale features essential for optical performance control. (**B**) The training dataset contains diverse metagrating geometries generated through our physics-constrained topology optimization approach. (**C**) DPRR histograms of metagratings produced from the trained PC-GAN generator, training set geometrically stretched for the design refractive index and period, and randomly generated binary patterns. The magnified view (inset) highlights the superior concentration of high-DPRR (0.2–0.8) designs from our approach. (**D**) Performance enhancement through topological refinement, showing DPRR distributions for the top 50 designs from both PC-GAN and training set after 100 optimization iterations. The refined designs demonstrate a 16% improvement in DPRR compared to initial configurations. (**E**) The DPRR performance curves for three distinct grating configurations: conventional gratings, PC-GAN-optimized gratings, and topologically refined gratings. (**F**) The DPRR distribution map of SLMG generated by the PC-GAN generator under different wavelengths and deflection angle parameters. (**G**) The representative DPRR distribution of the metasurface grating designed using pure iterative optimization (red histogram) and the retrained PC-GAN generated through topological optimization (blue histogram). The highest DPRR values are indicated by red and blue numbers. (**H**) The time cost of generating n metasurface gratings that meet three physical constraints using only iterative optimization (red line) and PC-GAN generation and refinement (blue line).

The PC-GAN framework demonstrates its unique capability through the autonomous discovery of optimized snowflake-like grating geometries (Fig. 3A), which exhibit superior uniformity compared to conventional diffraction gratings while maintaining comparable brightness - a critical advancement for augmented reality waveguide applications (note S6). The training methodology involves a sophisticated two-stage process: first, topology optimization generates an extensive dataset of metagrating configurations with fixed nanostructure height while systematically varying refractive indices (1.7, 2.0, 2.6) and grating period combinations [(918, 530), (751, 434), (590, 341) nm] (Fig. 3B). Second, the adversarial training incorporates three fundamental physical constraints: (i) broadband (400–700 nm) and wide-angle (30°–80°) efficiency requirements for three key diffraction orders [(0, 2), (1, 1), (−1, 1)], (ii) the DPRR for uniformity control, and (iii) the PCT used to address dispersion. These constraints are embedded directly into the generator’s loss function through differentiable physical layers, while the discriminator employs a dual evaluation strategy assessing both structural authenticity (via convolutional neural networks) and physical feasibility (through numerical verification of optical constraints). This innovative approach enables efficient exploration of the high-dimensional design space while guaranteeing all generated designs satisfy the stringent requirements for high-performance AR waveguide applications, including rigorous efficiency, uniformity, and chromatic dispersion targets.

To demonstrate the generalization capability of our conditional PC-GAN, we employ the trained generator to synthesize 2500 distinct metagrating configurations optimized for refractive indices 1.7 and grating period (918, 530) nm. The network demonstrates remarkable computational efficiency, generating thousands of viable designs in seconds–a throughput exceeding the original training dataset size with minimal computational overhead. We evaluate these designs using rigorous coupled-wave analysis (RCWA), with the resulting DPRR distribution presented in Fig. 3C. The histogram reveals a wide DPRR distribution spanning from 0 to 1, with a significant population of devices achieving near-effective DPRR values between 0.2–0.8. Particularly noteworthy is that DPRR values of optimal designs can exceed 0.9 and reach up to 0.95, demonstrating the network’s ability to extract and generalize fundamental physical principles from the training data. This generalization is enabled by the shared underlying wave optics governing light-matter interactions across this parameter space, which manifests in consistent topological features of high-performance devices. For applications requiring different material systems (e.g., alternative refractive indices), the framework maintains its effectiveness provided the training data encompasses the relevant physical parameter ranges.

The high-performance metagrating designs generated by our PC-GAN can undergo further refinement through iterative topology optimization to enhance several critical aspects. This post-processing stage primarily serves to: (i) optimize the DPRR closer to the ideal unity value, (ii) incorporate tolerance to nanofabrication imperfections such as feature size variations and sidewall angle deviations, and (iii) enforce practical manufacturing constraints including smaller grid snapping and 30 nm minimum feature sizes. Notably, this refinement process typically requires fewer than 100 optimization iterations since the PC-GAN-generated designs already exhibit near-ideal DPRR performance and reside in favorable local minima of the design space, as shown in Fig. 3D. Figure 3E presents a comparative analysis of DPRR performance curves for three distinct grating configurations: conventional gratings, PC-GAN-optimized gratings, and topologically refined gratings. The results demonstrate a progressive enhancement in optical uniformity, with our complete optimization framework achieving near-ideal DPRR values approaching unity–representing a substantial improvement over conventional design. This visual comparison provides compelling evidence for the effectiveness of our approach in addressing the critical uniformity challenges in waveguide-based AR displays.

Our PC-GAN framework achieves enhanced performance by iteratively incorporating optimized metagrating designs into its training refinement process. This improvement is achieved via two complementary data generation pathways: (i) conventional topology optimization, which established our initial training dataset, and (ii) PC-GAN-generated designs subsequently refined through physical-constrained optimization. The latter approach proves particularly computationally efficient for expanding the design space coverage. As a demonstration, we employed this dual approach to generate multiple additional high-performance metagrating designs (DPRR >0.8, the main three diffraction orders conform to the standard) spanning the complete parameter space. These designs were combined with the original training set to develop a second-generation PC-GAN. Figure 3F presents the DPRR distributions for SLMG at representative wavelength-angle combinations. The results demonstrate substantial improvement, with the retrained model producing designs that achieve DPRR values within 0.8–1 for over 80% of the parameter space, representing a 16% increase in design quality compared to the initial model. This iterative refinement process effectively creates a self-improving design framework where each generation of PC-GAN produces better-performing metagratings that can be used to train subsequent, more capable models.

Figure 3G presents the DPRR distributions of metagrating designs generated by our second-generation PC-GAN and subsequently refined through 100 iterations of topology optimization, compared against designs obtained through conventional topology-only optimization. The results demonstrate that for 73% of parameter combinations, the topologically refined PC-GAN designs achieve DPRR values comparable to or exceeding those of topology-only optimized designs, with 25% of cases showing superior performance. While the PC-GAN approach matches conventional optimization for high-refractive-index materials (*n* > 2.0) and small-period gratings (<500 nm), its performance shows room for improvement for low-index materials (*n* < 1.9) with larger periods (>800 nm). Several enhancement pathways are identified: (i) implementing deeper network architectures like ResNet with progressive resolution training, (ii) optimizing training parameter selection strategies, (iii) incorporating additional electromagnetic constraints into the adversarial framework, and (iv) expanding the training dataset coverage. The computational efficiency analysis (Fig. 3H) reveals that our approach reduces the computational cost by approximately 5× compared to conventional topology optimization when generating “above-threshold” designs (DPRR > 0.8) across the full parameter space. This advantage stems from the “offline” training phase that amortizes computational costs over subsequent design generations. Once trained, the PC-GAN can rapidly produce viable metagrating designs for target specifications while maintaining strict adherence to physical constraints, establishing it as an efficient design tool for practical AR waveguide development.

### Demonstration of the single-layer waveguide display using SLMG

**Fig. 4.**
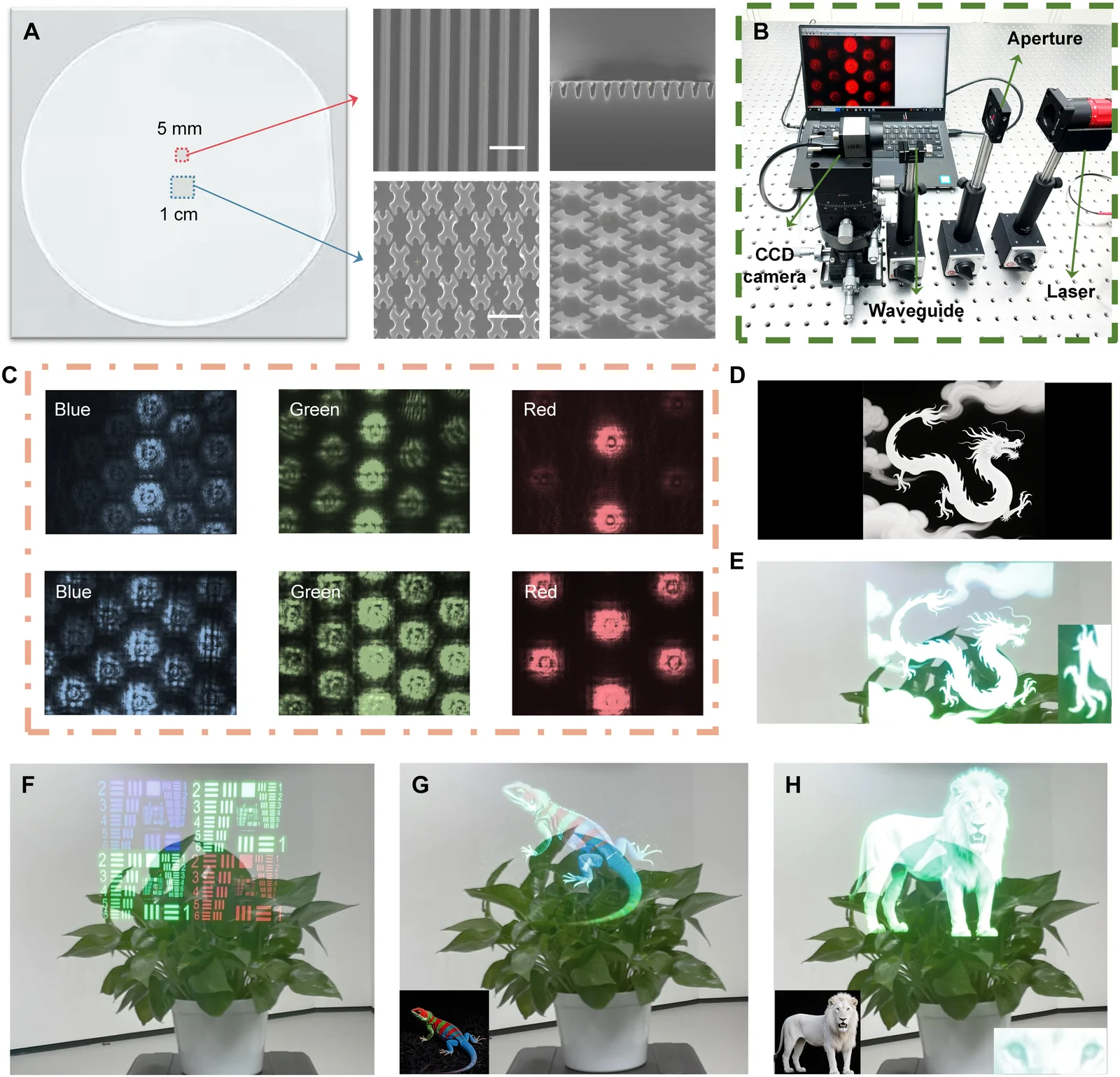
Demonstration of the single-layer waveguide display using SLMG. (**A**) Photograph of the fabricated metasurface waveguide with a thickness of 0.5 millimeters. Both IMC and OMC were prepared using polymers (*n* = 1.7). On the right are the scanning electron microscope (SEM) images of IMC and OMC. (**B**) The experimental optical path diagram used to measure the schematic diagram of pupil luminance uniformity. (**C**) The upper row is the experimental diagram of the RGB three-color exit pupil brightness uniformity of the traditional diffracted optical waveguide, and the lower row is the experimental diagram of the RGB three-color exit pupil brightness uniformity of the optical waveguide based on SLMG. (**D**) The original image for AR display. (**E**) Images taken in augmented reality mode. (**F** to **H**) Captured AR scenes. The plants and boxes behind the image are real objects, while the resolution board, lizards and lions are objects with superimposed numbers.

Figure 4A presents our fabricated single-layer waveguide system incorporating the SLMG as OMC (The specific processing parameters are provided in note S7). Due to fabrication constraints, we employed a nanoimprinting process to replicate the silicon master template into polymer waveguides (*n* = 1.7), with coupler dimensions of 0.5 cm × 0.5 cm (IMC) and 1 cm × 1 cm (OMC). To quantitatively evaluate the pupil expansion uniformity, we conducted laser-based characterization by injecting collimated RGB laser beams (λ = 460/532/620 nm) into the waveguide and imaging the output intensity distribution (Fig. 4B). The CCD-captured results in Fig. 4C reveal striking performance differences: conventional diffractive waveguides exhibit pronounced fishbone patterns (periodic luminance non-uniformity), while our SLMG waveguide maintains exceptional uniformity across all three wavelengths, including the typically problematic blue channel. Due to the limited CCD field-of-view, note S8 provides complete output pupil characterization, demonstrating consistent uniformity across the entire eyebox - a critical requirement for practical AR applications.

The imaging capabilities of our SLMG-based waveguide are comprehensively evaluated in Fig. 4, D to H, demonstrating exceptional performance across various illumination conditions. Figure 4E displays the white-light imaging results, revealing remarkable color uniformity. Figure 4H presents a particularly compelling demonstration of augmented reality functionality, showcasing a white lion virtual image seamlessly superimposed on real-world foliage. The preserved transparency and natural color rendering of the background leaves (visible through the lion’s lower body) confirm the waveguide’s excellent optical properties. These results collectively demonstrate that our SLMG waveguide successfully addresses the longstanding challenges of color fidelity, image uniformity, and real-world integration in AR systems.

### Demonstration of the single-layer waveguide display using elliptical-like metagrating (ELMG)

**Fig. 5.**
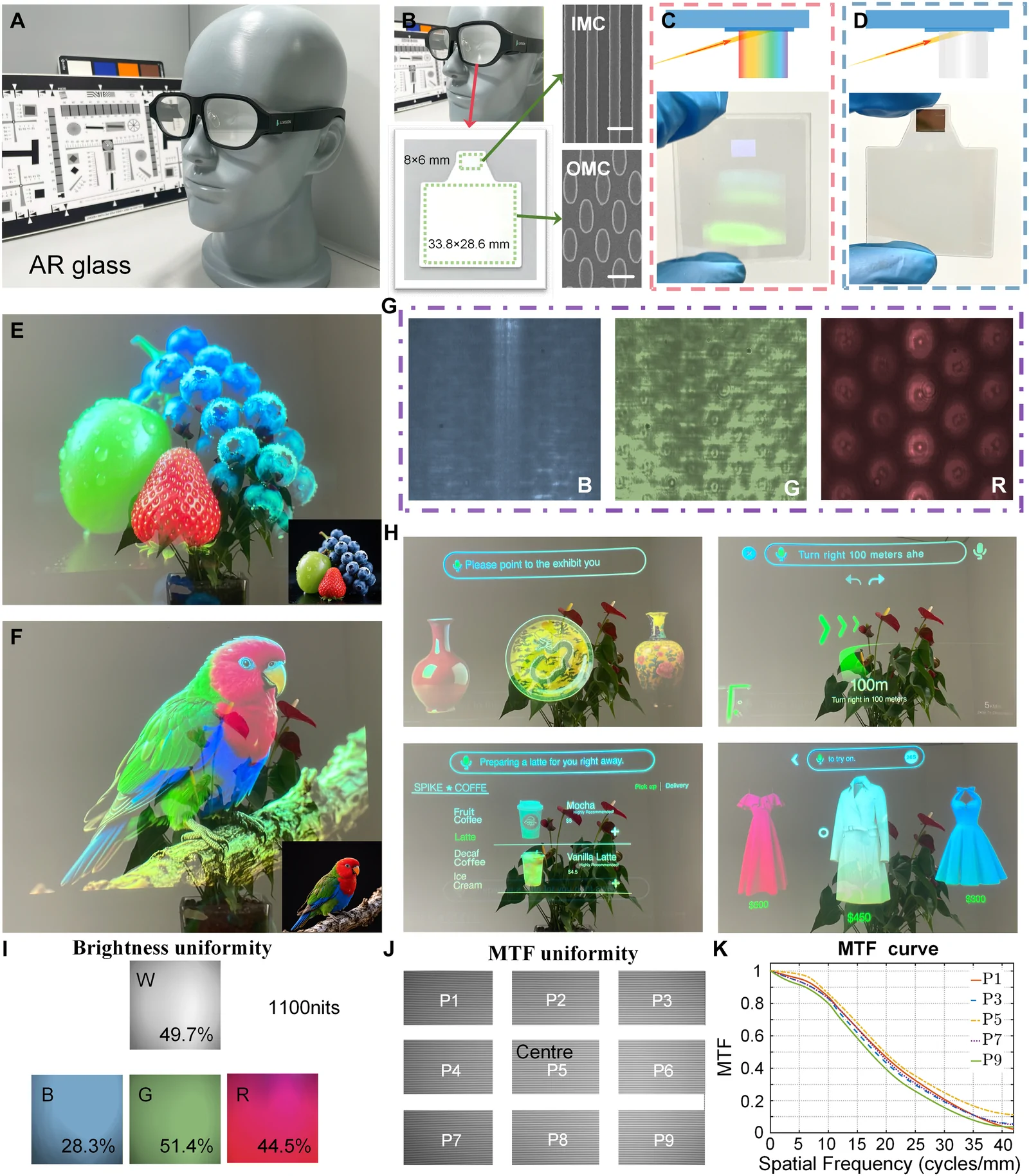
Demonstration of the single-layer waveguide display using ELMG. (**A**) Photograph of the assembled ELMG waveguide prototype. (**B**) Micrograph of the fabricated gratings showing the IMC (8 × 6 mm) and OMC (33.8 × 28.6 mm) patterned on a silicon carbide (SiC) substrate. Comparison of ambient light reflections between (**C**) conventional diffractive waveguide showing pronounced rainbow effect and (**D**) ELMG waveguide demonstrating artifact-free performance, with corresponding ray-tracing schematics illustrating the anti-dispersive design principle. (**E** and **F**) High-fidelity augmented reality imaging results showing full-color virtual content with preserved shadow details and clear transmission effects, validating the waveguide’s excellent image reproduction capability. (**G**) Measured output pupil intensity distributions for RGB (460/532/620 nm) illumination. (**H**) Practical application demonstrations including auction preview, navigation interface, and virtual shopping, highlighting the system’s real-world usability. (**I**) White light and RGB channel intensity uniformity mapping. (**J**) Spatial modulation transfer function (MTF) distribution. (**K**) Five field MTF curves.

While the SLMG demonstrates exceptional optical performance on high-refractive-index silicon carbide (SiC, *n* = 2.6) platforms, its intricate nanostructures pose significant fabrication challenges for current manufacturing processes. Figure 5A demonstrates our full-color AR display system employing PC-GAN-optimized ELMG, which achieve breakthrough performance in a compact single-layer architecture while significantly improving manufacturability (note S9). The prototype (Fig. 5B) incorporates precisely fabricated couplers with dimensions of 8 × 6 mm (IMC) and 33.8 × 28.6 mm (OMC) on a silicon carbide (SiC, *n* = 2.6) substrate, maintaining optical see-through capability. The waveguide’s anti-dispersion design is demonstrated in Fig. 5, C and D, showing complete elimination of rainbow effect under ambient illumination compared to conventional diffractive waveguides. Quantitative uniformity measurements (Fig. 5G) confirm consistent RGB intensity distribution, while Fig. 5, E and F showcase exceptional AR image quality with preserved fine details and realistic shadows. Practical application scenarios including auction previews, navigation cues, and virtual shopping are demonstrated in Fig. 5H.

Furthermore, when driven by a medium-intensity digital light processing (DLP Venus III) light engine under the same configuration, the waveguide achieved a notable brightness of 1100 nits, as supported by the brightness uniformity mapping in Fig. 5I which shows a white light uniformity of 49.7%. The calculation formula is $U=\left(1-\frac{P_{\max}-P_{\min}}{P_{\max}+P_{\min}}\right)\times 100\%$. This performance is maintained despite the compact 1 mm waveguide thickness, overcoming the traditional trade-off between optical performance and form factor. As demonstrated by the MTF analysis in Fig. 5, J and K, image quality is well preserved, with an average spatial frequency of 15 cycles per mm maintained at MTF >0.6. Additionally, the color consistency across the entire field of view is 0.102. The spatial uniformity of MTF demonstrates that our design avoids the localized resolution degradation characteristic of conventional pupil-expanding waveguides. The correlation between these measurements proves the ELMG architecture fundamentally addresses the interlinked challenges of the trilemma, rather than optimizing one parameter at the expense of others (note S10).

## DISCUSSION

This work presents a transformative solution to the fundamental efficiency-uniformity-dispersion trilemma that has long constrained augmented reality waveguide displays, achieved through the synergistic integration of physics-constrained generative design and innovative metagrating architectures (note S11). Our developed PC-GAN framework fundamentally rethinks nanophotonic inverse design by embedding first-principles optical constraints directly into the generative process, enabling the discovery of non-intuitive SLMG and ELMG that simultaneously achieve arbitrary control of three diffraction orders across the visible spectrum, excellent uniformity, and exceptional dispersion control in compact single-layer waveguides. The key innovation lies in establishing rigorous physical metrics–particularly the DPRR ≈ 1 and PCT–that effectively decouple traditionally competing performance parameters, allowing the system to navigate previously inaccessible regions of the design space. Experimental validation confirms these computational designs translate successfully to fabricated components, with silicon carbide (*n* = 2.6) implementations demonstrating smaller luminance variation, excellent color fidelity, and MTF > 0.6. The 75° ultrawide FOV of the SiC waveguide relies on the synergy between the high refractive index of SiC (the foundational material prerequisite) and the PC-GAN-generated metagrating design (the key structural guarantee for uniform outcoupling efficiency and artifact suppression across the entire FOV). Meanwhile, these devices eliminate rainbow effect and fishbone patterns that plague conventional approaches. The SLMG demonstrate superior performance in controlling multi-order diffraction and achieving wide angular response uniformity, while the ELMG offer a more fabrication-tolerant alternative with slightly compromised optical performance (0.2 reduced DPRR compared to SLMG)– together establishing a versatile design portfolio for diverse manufacturing scenarios. Specifically, the ELMG serve as a practical fallback solution for production lines with less mature nanofabrication capabilities, providing a viable performance-to-manufacturability tradeoff without requiring advanced patterning techniques. This dual-architecture approach ensures technology deployability across different industrial contexts while maintaining essential AR display specifications.

Looking forward, this physics-constrained generative approach establishes a versatile platform that can be extended along several transformative directions. The immediate pathway involves scaling the framework to full-color, wide-FOV systems through dynamic resolution progressive training and 3D meta-atom optimization while incorporating additional multi-physics constraints for thermal and mechanical robustness. Beyond display applications, the fundamental methodology shows promise for revolutionizing other nanophotonic systems where multi-parameter optimization is crucial, including ultra-compact spectrometers, quantum optical circuits, and biomedical sensing interfaces. The demonstrated success in bridging computational design with mass-production via nanoimprint lithography suggests near-term potential for commercial translation, particularly as the field progresses toward thinner form factors and higher performance requirements that demand such rigorous physical constraints. More broadly, this work provides a template for merging deep learning with fundamental physics across disciplines–a paradigm that will grow increasingly vital as we approach the limits of conventional design methodologies in advanced optical systems. The integration of real-time experimental feedback and expanded constraint libraries promises to further enhance the framework’s capabilities, potentially enabling autonomous laboratories for photonic device development. As augmented reality evolves toward ubiquitous computing platforms, the solutions developed here for overcoming the core trilemma will prove foundational in achieving the necessary combination of military-grade optical performance, consumer-electronics aesthetics, and mass-manufacturing scalability required for widespread adoption.

## MATERIALS AND METHODS

### SLMG fabrication

The fabrication of the SLMG started from the spin coating of the resist (PMMA 950 A5) on a Si substrate, where 300 nm resist was obtained by a spin speed of 4000 rpm. Electron beam lithography (Raith EBPG 5200) was then performed to write the pattern under the write voltage of 100 kV with a dose of 750 μC/cm^2^. Subsequently, the process of the PMMA was executed by immersing the sample into the MIBK: IPA = 1:3 solution for 40 s, followed by 100 nm Cr deposition by the E-beam deposition. Then, the sample was immersed for 20 h for the lift-off, and the snowflake-like Cr pattern was formed as the etching mask. An inductively coupled plasma reactive ion etching (ICP) was utilized to etch the Si to the target depth. Finally, the Si nanostructures were realized after removing the residual Cr by remover of ammonium cerium nitrate and perchloric acid solution (note S12). To fabricate the final waveguide, the Si nanostructure served as a master template for nanoimprint lithography (NIL). A UV-curable resin was pressed onto the template under optimized pressure and temperature, then cured with UV exposure. The replicated polymer waveguide retained the precise snowflake-like grating structures, enabling high-fidelity light manipulation while maintaining the SLMG’s hierarchical optical properties.

### ELMG fabrication

Our SiC waveguide AR glasses integrate a high-refractive-index SiC waveguide, fabricated through an optimized NIL process. Unlike conventional diffractive waveguides that rely on soft resist materials prone to damage, we used a robust NIL-to-lift-off process to enhance durability and optical performance. This method replaced traditional NIL-to-etch techniques, which suffer from rapid resist degradation during etching, with a metal hard mask (Cr) that improves etch selectivity and minimizes defects. The process began with high-precision electron beam lithography (EBL) on a silicon master (Raith EBPG, 100 kV, 10 nA exposure), followed by inductively coupled plasma (ICP) etching to achieve deep, high-fidelity grating structures. To extend mold longevity, the pattern was transferred to a PET film via UV-curing and anti-adhesion treatment, enabling repeated imprinting without degradation (note S13).

### Numerical simulations

Our PC-GAN framework employs a carefully designed architecture where the generator processes a latent vector concatenated with refractive index and period inputs to produce fabrication-ready metagrating patterns on a 1000 × 800 grid. To ensure manufacturability, we integrate a Gaussian smoothing layer that eliminates sub-30 nm features while preserving critical optical characteristics. The network is trained on 600 topology-optimized valid samples using PC-GAN with gradient penalty and Adam optimization (α = 0.001), converging within 5–10 minutes on the NVIDIA GeForce RTX 3090. A hybrid discriminator architecture uniquely combines conventional pattern recognition with real-time physical validation through an embedded RCWA solver, enforcing strict compliance with our core constraints: DPRR ≈ 1, PCT, and the efficiency of the three reasonable diffraction orders.
