# Supplementary material for: Ultrawide-FOV full-color waveguides free of rainbow and fishbone artifacts via physics-constrained generative metagrating design
Source: Sci Adv. 2026 Aug 19;12(34):eaeh5234. doi: 10.1126/sciadv.aeh5234 (PMC13488895; doi:10.1126/sciadv.aeh5234)
Supplement: Supplementary file 1 [file sciadv.aeh5234_sm.pdf]

Science Advances
AAAS

Supplementary Materials for

Ultrawide-FOV full-color waveguides free of rainbow and fishbone artifacts via physics-constrained generative metagrating design

Mengguang Wang *et al.*

Corresponding author: Zhenrong Zheng, zzr@zju.edu.cn

Sci. Adv. **12**, eaeh5234 (2026)
DOI: 10.1126/sciadv.aeh5234

This PDF file includes:

Supplementary Text
Figs. S1 to S26
Tables S1 and S2

S1. Analysis of the pupil dilation process of two-dimensional optical waveguides

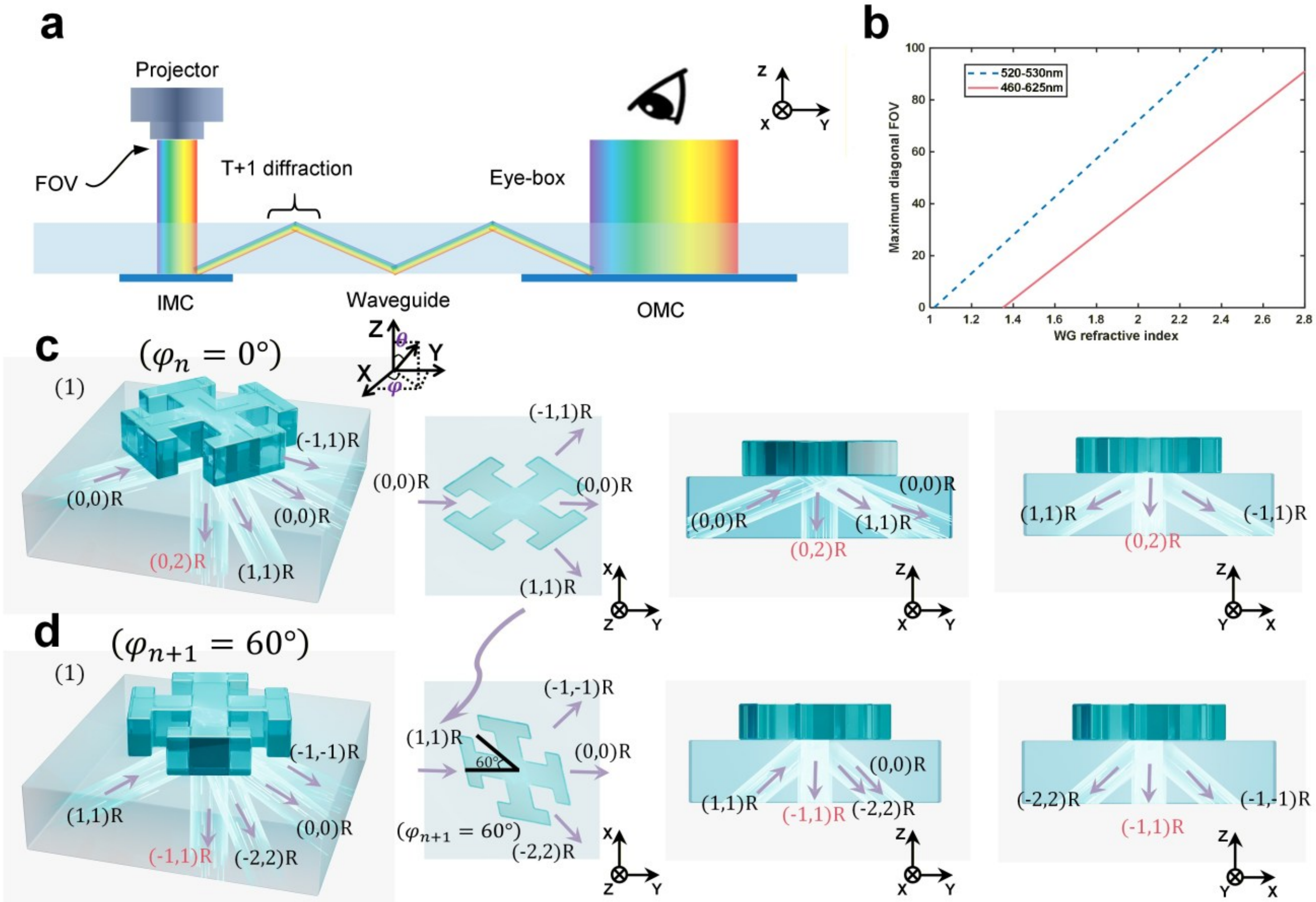

Figure S1. Analysis of the pupil dilation process of two-dimensional optical waveguides. (a) Schematic illustration of the optical path comprising components such as the micro-display and waveguide, depicting the diffraction and reflection processes of RGB light within the waveguide. (b) Calculated maximum FOV as a function of refractive index for both the monochromatic and visible light bands. (c) The diffraction situation of the metasurface grating when the φ_n azimuth angle is 0°. (d) The diffraction situation of the metasurface grating when the φ_{n+1} azimuth angle is 60°.

Figure S1(a) presents the cross-sectional schematic of the waveguide architecture, illustrating the light propagation mechanism between the input and output metasurface couplers. The corresponding FOV dependence on refractive index is quantitatively characterized in Fig. S1(b), revealing a linear relationship critical for wide-angle operation.

More importantly, as clearly demonstrated in Fig. S1(c) and S1(d), the coupling dynamics during the two primary diffraction processes reveal a fundamental transition from an outcoupling configuration dominated by orders (0, 2) R to one governed by orders (-1, 1) R. This shift directly elucidates the origin of the fishbone-like striations observed in conventional designs, which arise from inefficient and asymmetric energy distribution. To address this, we introduced dilated pupil restraint ratio (DPRR)—an innovative approach that actively adjusts the magnitudes of the coupling levels in different directions to suppress unnecessary diffraction levels, while enhancing energy uniformity across the output plane. The result is a remarkable improvement in optical field homogeneity, underscoring the effectiveness and novelty of our DPRR concept in achieving high-performance waveguide displays.

S2. Comparison of the DPRR distribution between traditional diffraction gratings and SLMG

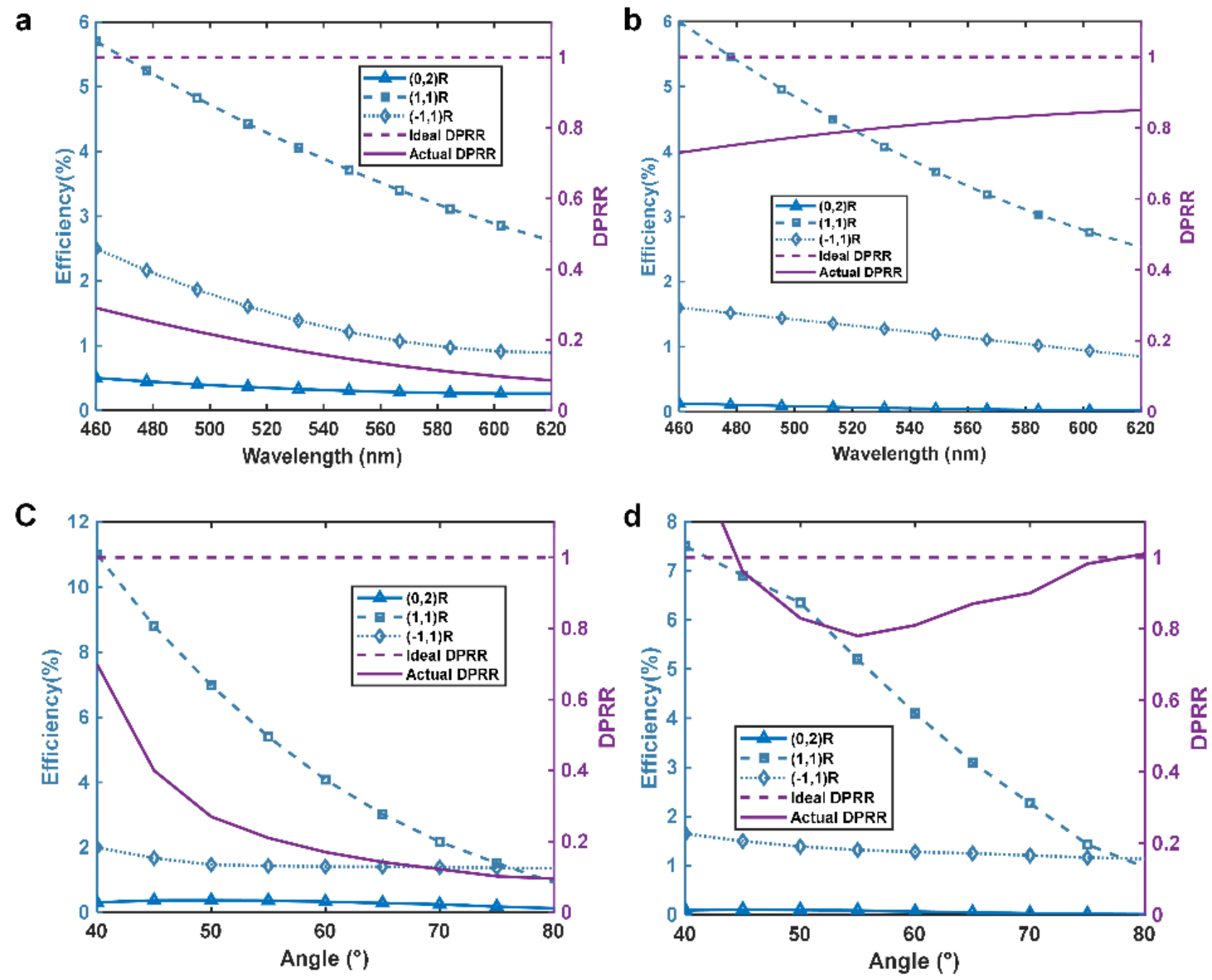

Figure S2. A comprehensive comparison of diffraction efficiency and DPRR distributions between conventional diffractive gratings and SLMG under varying operational conditions. (a) The wavelength uniformity of traditional diffraction gratings. (b) The wavelength uniformity of SLMG. (c) The angular uniformity of traditional diffraction gratings. (d) The angular uniformity of SLMG.

Figure S2 presents a comprehensive comparison of diffraction efficiency and DPRR distributions between conventional diffractive gratings and SLMG under varying operational conditions. The wavelength-dependent performance (460–620 nm) for three dominant diffraction orders [(0, 2), (1, 1), (-1, 1)] is shown in Figs. S2(a) and S2(b), where SLMGs demonstrate significantly improved efficiency uniformity and DPRR values compared to conventional gratings. Figs. S2(c) and S2(d) further analyze angular responses (40–80°), revealing that SLMGs maintain stable efficiency and DPRR across the entire field-of-view, while conventional designs exhibit severe DPRR deviations. These results quantitatively validate SLMG's superior capability in simultaneously addressing chromatic dispersion and angular uniformity challenges in waveguide

displays. The (0, 2) diffraction orders of our optimized SLMG are intentionally kept relatively low. This design is aimed at reducing the attenuation rate and improving the uniformity, which are essential requirements for achieving a wide field of view and a big eyebox. More importantly, this does not compromise the final brightness: under the same conditions, the brightness of our waveguide reaches 1100 nits, whereas that of the comparative diffraction waveguide is only around 1000 nits.

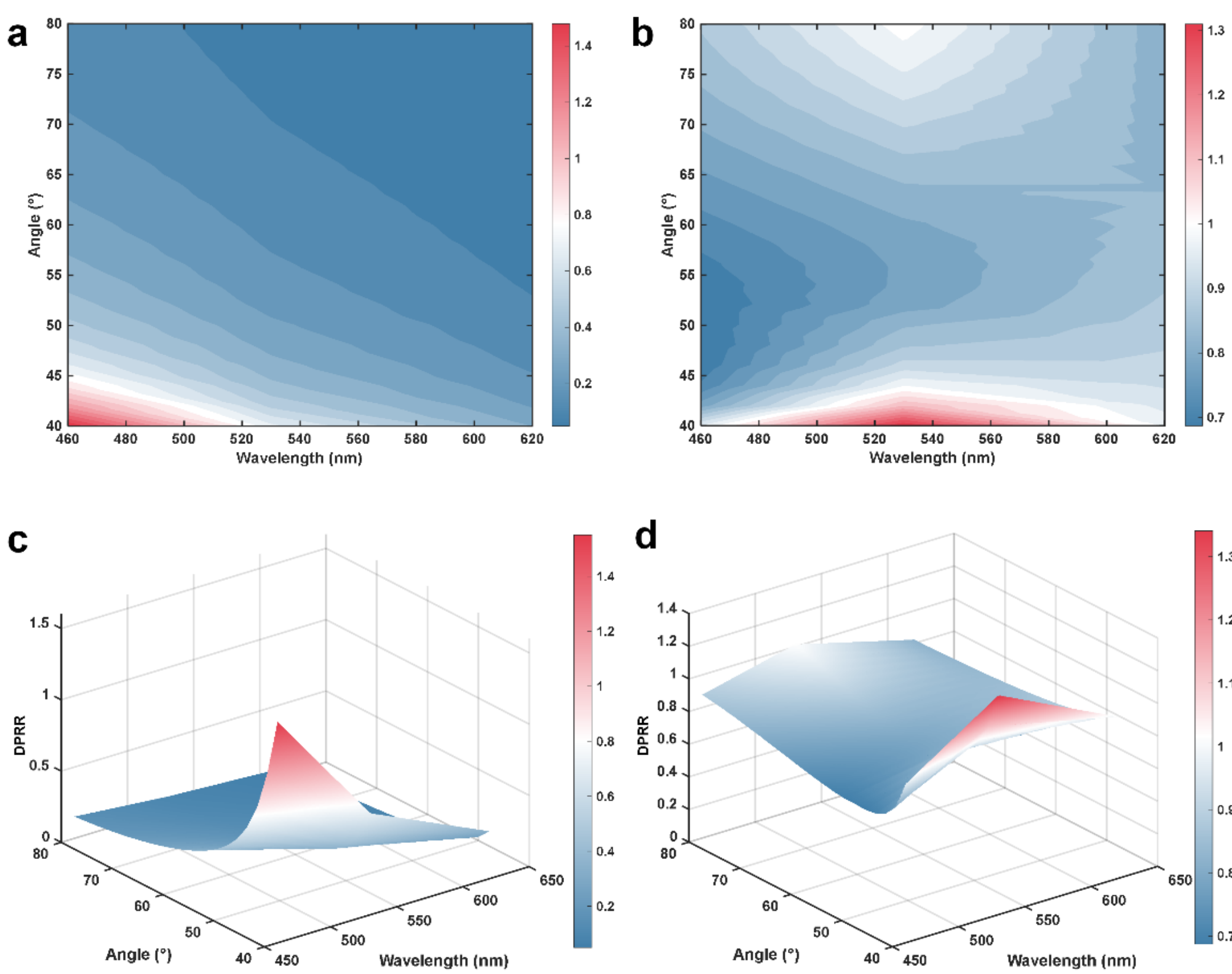

Figure S3. A comprehensive comparison of DPRR distributions between conventional diffractive gratings and SLMG under varying operational conditions. (a) The DPRR distribution of traditional diffraction gratings with respect to wavelength and angle. (b) The DPRR distribution of SLMG with respect to wavelength and angle. (c) Schematic diagram of the three-dimensional distribution of DPRR of traditional diffraction gratings with respect to wavelength and angle. (d) Schematic diagram of the three-dimensional distribution of DPRR of SLMG with respect to wavelength and angle.

S3. Dispersion analysis

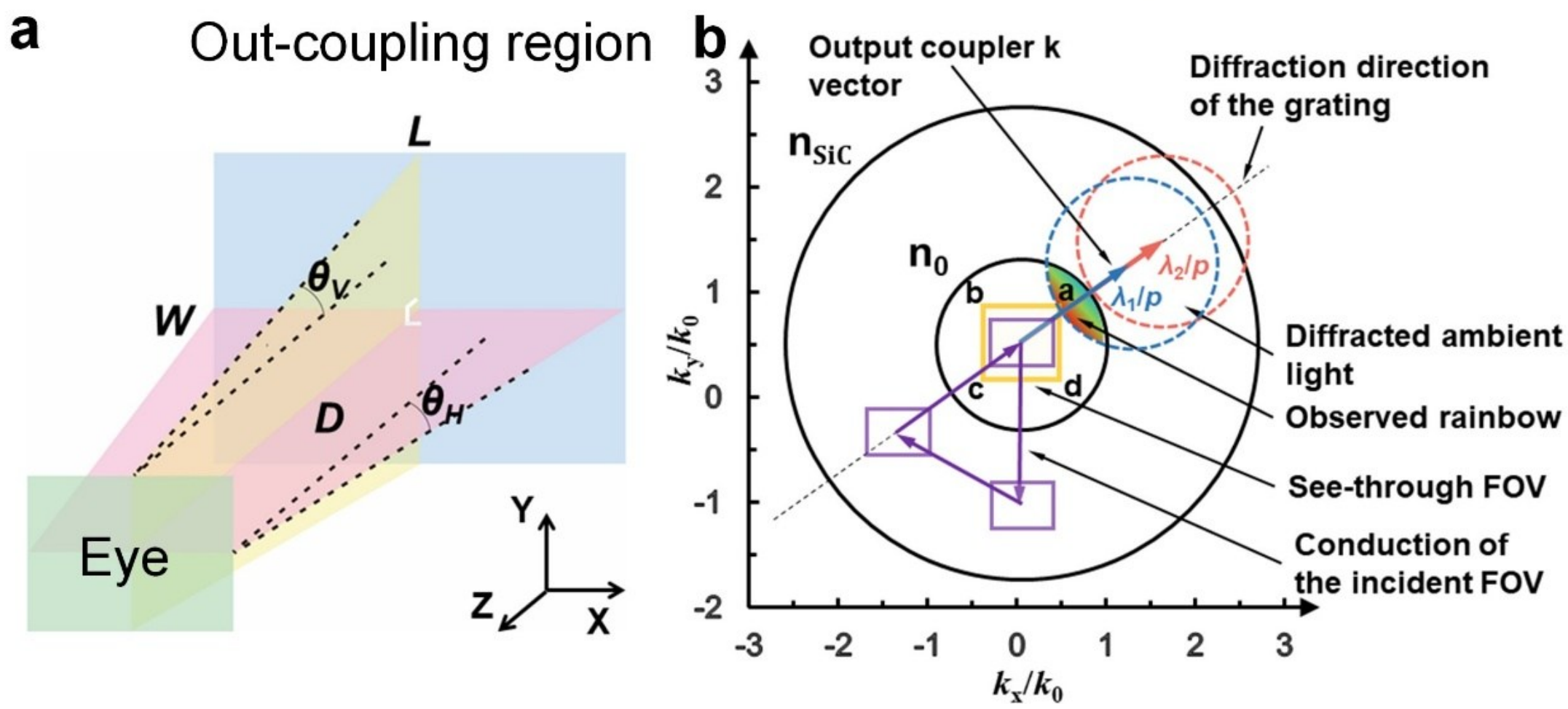

Figure S4. Dispersion analysis. (a) illustrates the geometric configuration of the waveguide exit pupil (output coupler) region, specifying the horizontal (θ_H) and vertical (θ_V) viewing angles relative to the observer's position, where *D* denotes the eye relief distance to the output coupling region. The corresponding *k*-space representation in (b) depicts the light propagation characteristics, with the inner and outer circles representing the normalized wavevectors in air and SiC (n=2.6) respectively. Wavelength-dependent dispersion is indicated by the shifted blue (450 nm) and red (650 nm) annular regions, offset by λ_i/p along the grating's diffraction axis (where λ_i is the incident wavelength and p the grating period). The rectangular boundary marks the wavevector range corresponding to the system's field-of-view, while the input and output grating vectors are oriented at 0° and 60° relative to the reference axis, with all wavevectors normalized to $2\pi/\lambda$.

In augmented reality waveguide displays, the chromatic dispersion artifact represents a critical challenge that significantly degrades the viewing experience. This issue arises from spectral splitting caused by the diffractive phenomenon of ambient illumination through the output grating coupler when external light directly impinges on the grating emission region. To tackle this problem and streamline the design methodology for diffractive waveguides, we introduce a novel design framework: the periodic constraint theory (PCT). This approach enables more

intuitive and efficient refinement of grating parameters, mitigating the impact of chromatic aberration while enhancing the overall performance of the display system.

Given that the light propagation mechanism of the waveguide's output grating mirrors that of the input grating, only higher-order diffracted beams are likely to couple out of the waveguide and contribute to human visual imaging. Taking the waveguide interface normal as a reference, consider the first-order diffracted light as an example; the grating equation can be articulated as

$$n\, sin\, \theta_{-1} = n_0 \cdot sin\, \theta_{in} + \frac{\lambda_0}{p} \tag{S1}$$

In the formula, the incident angle is θ_{in}, the diffraction angle of the -1-order is θ_{-1}, the refractive index of the incident medium air n_0=1, the refractive index of the waveguide medium is denoted as n, and p is the grating period. Obviously, for light transmission, TIR must occur (i.e., $\theta_{-1} > \theta_c = \arcsin(1/n)$), and $\theta_{-1} < 90°$, and the working wavelength λ_0 of the waveguide is in the RGB band. Based on the above boundary conditions of the diffractive waveguide, the following can be obtained as

$$\frac{\lambda_{Red}}{n - sin\theta_{in}} < p \leq \frac{\lambda_{Blue}}{1 - sin\theta_{in}} \tag{S2}$$

where λ_{Red} is the maximum wavelength of the light beam emitted by the optomechanical device, usually 650 nm for red light, and λ_{Blue} is the characteristic wavelength of blue light in natural light, usually 450 nm. Equation (S2) indicates that the grating period is related to the field-of-view (FOV) angle range of the optomechanical device, the refractive index of the waveguide, and the size of the outcoupling area.

Figure S4(a) depicts the real-space parameters of the waveguide in the exit pupil area, where the waveguide length is L, the width is W, the observation distance of the human eye is D, the horizontal FOV angle is θ_H, and the vertical FOV angle is θ_V. In the k-diagram, the boundaries of

the exit pupil area in the real space are transformed into a rectangular region with four vertices a, b, c, and d. Their coordinates are $(L/\sqrt{4D^2+L^2}, W/\sqrt{4D^2+W^2})$, $(-L/\sqrt{4D^2+L^2}, W/\sqrt{4D^2+W^2})$, $(-L/\sqrt{4D^2+L^2}, -W/\sqrt{4D^2+W^2})$, and $(L/\sqrt{4D^2+L^2}, -W/\sqrt{4D^2+W^2})$ respectively, as shown in Fig. S4(b). Considering that diffracted ambient light with a wavelength of λ is obliquely incident on the exit pupil area, it is equivalent to an inner circle with a radius of 1 moving λ/p along the direction of the outcoupling grating vector, where p is the period of the exit pupil grating. At this time, if they moved circle overlaps with the see-through FOV area, rainbow patterns may be observed in the corresponding area of the waveguide. To ensure that there is no rainbow diffraction within the entire wavelength band, combined with the total reflection relationship, the grating period p needs to satisfy

$$\frac{\lambda_{\mathrm{Red}}}{n-\sin\theta_{\mathrm{in}}} < p \leq \frac{\lambda_{\mathrm{Blue}}}{1+\sin\theta_{\mathrm{max}}} \quad \text{(S3)}$$

where θ_{max} is the maximum angle between the input light ray and the normal direction of the plane where the waveguide substrate is located, that is, the maximum viewing angle of the human eye. It can be derived from the formula $\tan\theta_{\mathrm{max}} = L/2D$, where L is the length of the outcoupling area and D is the size of the exit pupil distance.

S4. The representation of rainbow effect

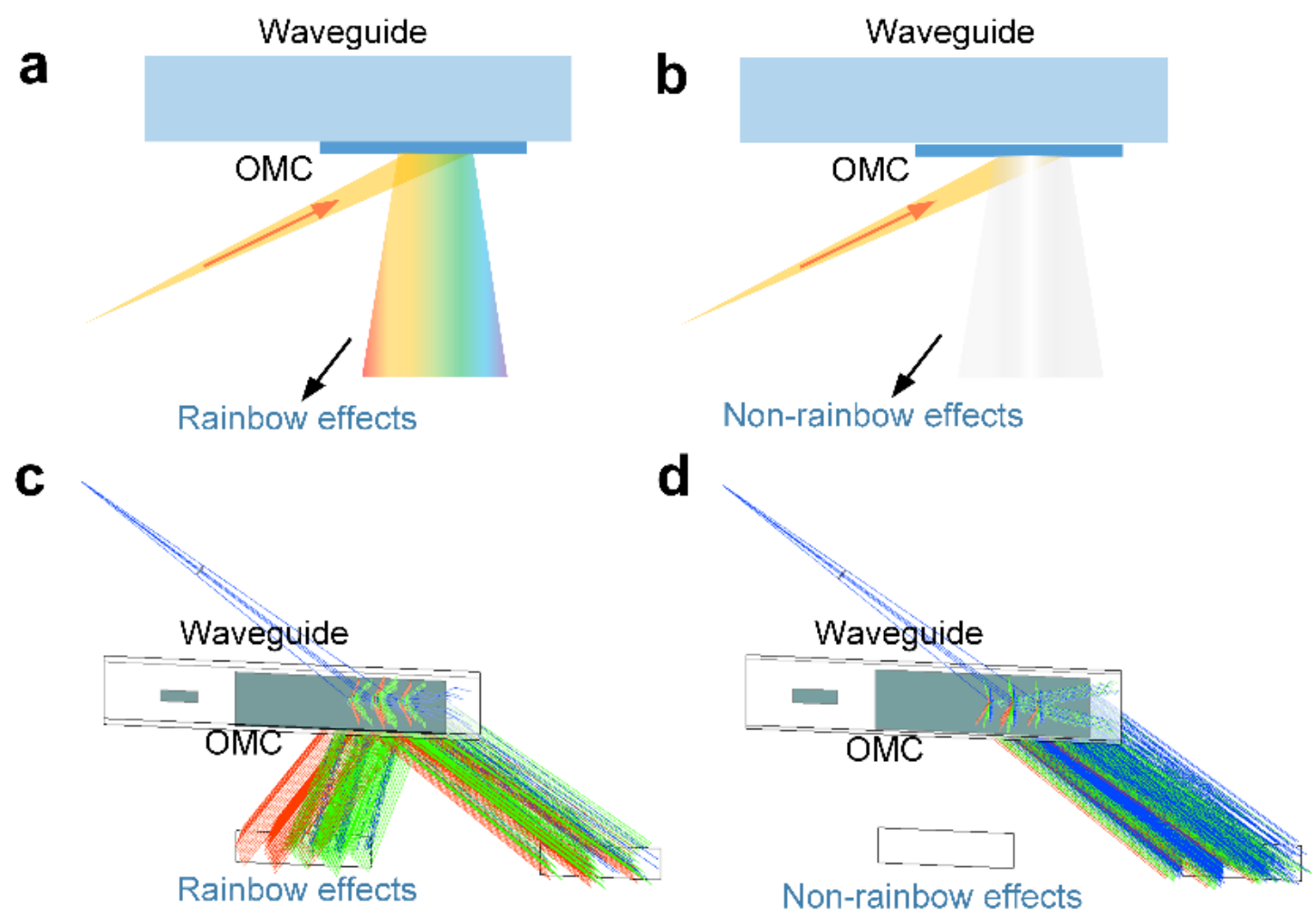

Figure S5. The representation of rainbow effect. (a) and (b) Schematic diagrams of the optical paths for the generation of reflected rainbow patterns and non-reflected rainbow patterns by ambient light. (c) Dispersion characterization of traditional diffraction gratings. (d) Dispersion characterization of SLMG.

To quantitatively evaluate chromatic dispersion effects, we employed Zemax optical simulations to analyze conventional diffractive gratings, with results shown in Fig. S5(c). The simulations clearly demonstrate pronounced rainbow artifacts caused by wavelength-dependent diffraction, particularly under oblique ambient illumination.

In contrast, Figure S5(d) validates the effectiveness of our proposed PCT theory, showcasing snowflake-like metagratings (SLMGs) designed under this framework. The simulations confirm complete elimination of rainbow effect while maintaining high diffraction efficiency across the visible spectrum (400–700 nm).

S5. The principle of reverse design accompanied by topology optimization

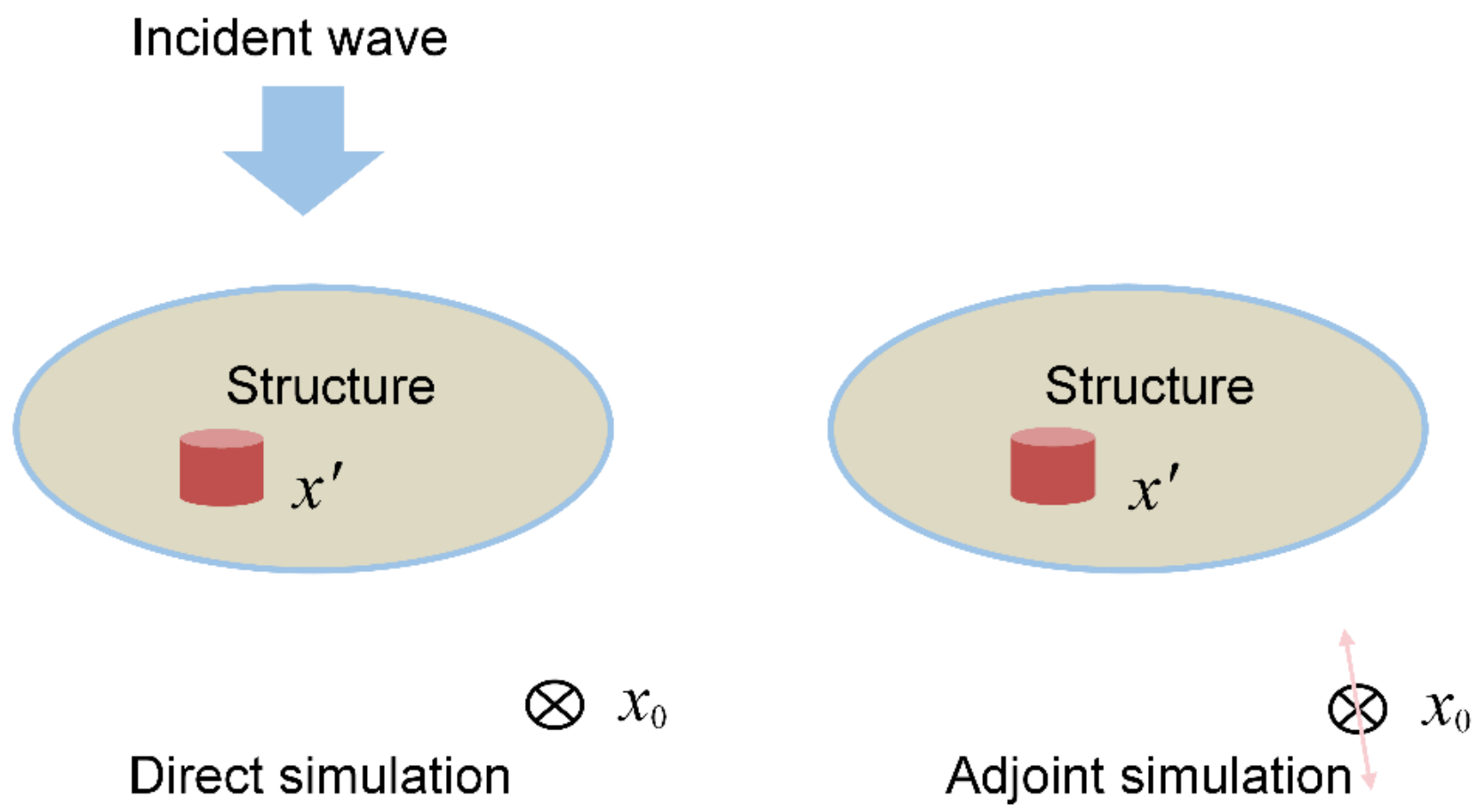

Figure S6. Schematic diagram of the principle of topology optimization.

Reverse design refers to first determining the required target response and then obtaining the structure through algorithm optimization. Adjoint-based topology optimization is a reverse design method. After giving the ideal target response, a structure is randomly initialized. Then, the electromagnetic fields of the forward simulation and the adjoint simulation are calculated through electromagnetic simulation (RCWA, FDTD, FEM), thereby determining the gradient change of the evaluation function. Suppose there is a structure where light is incident from the top of the structure. For any point x_0 outside the structure, we want to maximize the electric field intensity at x_0. This can be represented by a function, that is $F = \frac{1}{2}|E(x_0)|^2$.

Now consider the influence of introducing a small dielectric with a radius of r and a dielectric constant of ε_2 at x'. The change of the evaluation function is merely the difference between the evaluation function of the new geometric shape and that of the old geometric shape:

$$\delta F = \frac{1}{2}\left|E^{\text{new}}(x_0)\right|^2 - \frac{1}{2}\left|E^{\text{old}}(x_0)\right|^2 \tag{S4}$$

For the electric field of the new geometric shape, it can be expressed as $E^{\text{new}}(x_0) = E^{\text{old}}(x_0) + \delta E(x_0)$, then

$$\delta F = \frac{1}{2}\left[E^{\text{old}}(x_0) + \delta E(x_0)\right]\overline{\left[E^{\text{old}}(x_0) + \delta E(x_0)\right]} - \frac{1}{2}E^{\text{old}}(x_0)\overline{E^{\text{old}}(x_0)}$$
$$= \frac{1}{2}\left[E^{\text{old}}(x_0)\overline{\delta E(x_0)} + \overline{E^{\text{old}}(x_0)}\delta E(x_0) + |\delta E(x_0)|^2\right] \quad \text{(S5)}$$
$$\approx \text{Re}\left[E^{\text{old}}(x_0)\delta E(x_0)\right]$$

Among them, the overbar denotes complex conjugation. The change $\delta E(x_0)$ of the electric field $E^{\text{old}}(x')$ at x_0 between the two geometric shapes is caused by the interaction of the dielectric inserted at x'. The electric field intensity of the original geometric shape at x' is $E^{\text{old}}(x')$. When a cylindrical dielectric is inserted, the effect is to add an induced dipole moment p^{ind}at x', with an amplitude of

$$p^{\text{ind}} \simeq \pi r^2(\epsilon_2 - \epsilon_1)E^{\text{old}}(x') \quad \text{(S6)}$$

The complete one is given by the product of Green's function and the induced dipole moment, that is $\delta E(x_0) = G(x_0, x')p^{\text{ind}}(x')$, then

$$\delta F = \text{Re}\left[\overline{E^{\text{old}}(x_0)}G(x_0, x')p^{\text{ind}}(x')\right] \quad \text{(S7)}$$

Now, the influence of the dielectric at x' on the evaluation function can be calculated through a single simulation. However, for the change of the dielectric constant at any point x on the material, a simulation is required to calculate the evaluation function.

If a single metasurface unit structure is divided into $n*m$ pixel points, and the dielectric constant of each pixel point can be a set material (such as silicon) or air, then corresponding to the formula, there are x'_1, x'_2... The dielectric constants of x'_{n*m} points will change. To calculate the impact of the simultaneous changes of these points on the evaluation function, according to the forward simulation formula (S8), $n*m$ forward simulation calculations are required.

Substitute the symmetry of Green's function into the equation. Then equation (S8) becomes

$$\delta F = \text{Re}\left[\overline{E^{\text{old}}(\text{x}_0)}G(\text{x}', \text{x}_0)p^{\text{ind}}(\text{x}')\right] = \text{Re}\left[G(\text{x}', \text{x}_0)\overline{E^{\text{old}}(\text{x}_0)}p^{\text{ind}}(\text{x}')\right] \quad \text{(S8)}$$

For the first two terms of the equation, $G(x', x_0)\overline{E^{old_0}}$ is precisely the field of the dipole driven by the amplitude $\overline{E^{old_0}}$. This dipole is driven at x_0 and radiates to x'. If we define the accompanying electric field:

$$E^A(x') = G(x', x_0)\overline{E^{\text{old}}(x_0)} \tag{S9}$$

$$\delta F = Re\left[E^A(x')p^{\text{ind}}(x')\right] \tag{S10}$$

Two calculations were conducted. The field at x_0 was calculated in the forward simulation, and then the adjoint electric field at x' was calculated based on $E^A(x') = G(x', x_0)\overline{E^{\text{old}}(x_0)}$ in the adjoint simulation. Finally, the change of the evaluation function was expressed in terms of the adjoint electric field and the induced dipole moment.

Now, by introducing the accompanying electric field and taking the target x_0 as the dipole source, the electric field radiated by x_0 to each pixel point on the metasurface can be calculated at one time. Forward simulation can calculate the electric field at the target point x_0 after the dielectric constant of all points on the metasurface changes at one time.

With the introduction of the electric field, the forward electric field is calculated. By further calculating the associated electric field based on the forward electric field, the changes in the dielectric constant of all pixels on the metasurface and the systems between the evaluation functions can be obtained through a single electromagnetic simulation.

If the above situation is extended to a more general one, then

$$\delta F = 2\,Re\int_{\psi} P^{ind}(x') \cdot E^A(x')d^3x' \tag{S11}$$

For the topological optimization process, $P^{ind}(x) = \bar{\bar{\alpha}}E(x)$, $\bar{\bar{\alpha}}$ are shape-related polarizabilities, then

$$\delta F = 2\, Re \int_{\psi} \overline{\alpha}\, E(x') \cdot E^{A}(x') d^{3}x' \tag{S12}$$

The change in the electric field at x_0 caused by the introduction of a small dielectric at x' can be represented by a forward simulation electric field and an adjoint simulation electric field. The change in the electric field at x_0 caused by the change in the dielectric at any point x can be expressed by Equation (S13).

If the change δF of the evaluation function is set as the derivative of the dielectric constant ε of the pixel points on the metasurface, then the evaluation function can quickly reach the target value by the gradient descent method. In this way, the evaluation function can be linked to the variation of the dielectric constant at all pixel points on the metasurface. Our design goal can be linked to the dielectric constant of any shape distribution on the metasurface.

The Torcwa library is implemented using PyTorch and calculated on the GPU. It has the function of automatic differentiation, which greatly improves the calculation speed. We only need to focus on the change of the evaluation function FOM.

The refinement step adopts a multi-objective weighted loss function, with the core goal of ensuring both physical realizability and strict compliance with DPRR constraints, while maintaining the desired optical performance. The loss function is explicitly defined as a weighted combination of two key terms, with weights assigned based on their priority in achieving the design targets:

DPRR Constraint Term (weight = 0.7, highest priority): This term is the core of the loss function, defined as the absolute deviation between the actual DPRR value of the refined metagrating and the target value (DPRR $\approx$ 1), i.e., $\mathcal{L}_{\mathrm{DPRR}} = |\mathrm{DPRR}_{\mathrm{actual}} - 1|$. Maximizing the weight of this term ensures that the refinement process prioritizes meeting the DPRR constraint, which is critical for suppressing fishbone artifacts and ensuring brightness uniformity.

Diffraction Efficiency Deviation Term (weight = 0.3): This term focuses on the target optimization of the 27 key parameters in the output metasurface coupler (OMC) that are simultaneously optimized by our PC-GAN framework—specifically corresponding to the three key diffraction orders [(0, 2), (1, 1), (-1, 1)] for RGB wavelengths (460 nm, 530 nm, 620 nm) at three distinct incident angles (40°, 60°, 80°). It minimizes the deviation between the diffraction efficiency of the refined metagrating and the target efficiency (optimized during PC-GAN training) for each of these 27 key parameter combinations, i.e., $\mathcal{L}_{\text{eff}} = \sum\left|\eta_{\text{actual},i} - \eta_{\text{target},i}\right|$ (where i denotes each of the 27 key parameter combinations). This term guarantees that the post-processing refinement does not degrade the optical performance of the OMC, as it strictly maintains the target efficiency for all critical parameter configurations optimized by the PC-GAN.

The weight assignment is determined by the priority of design requirements: DPRR compliance (core constraint) > diffraction efficiency (target optimization of 27 key parameters, critical performance guarantee). This loss function ensures that the refined metagrating not only meets the DPRR and PCT constraints but also maintains the optimized optical performance of the 27 key OMC parameters.

S6. The result of PC-GAN

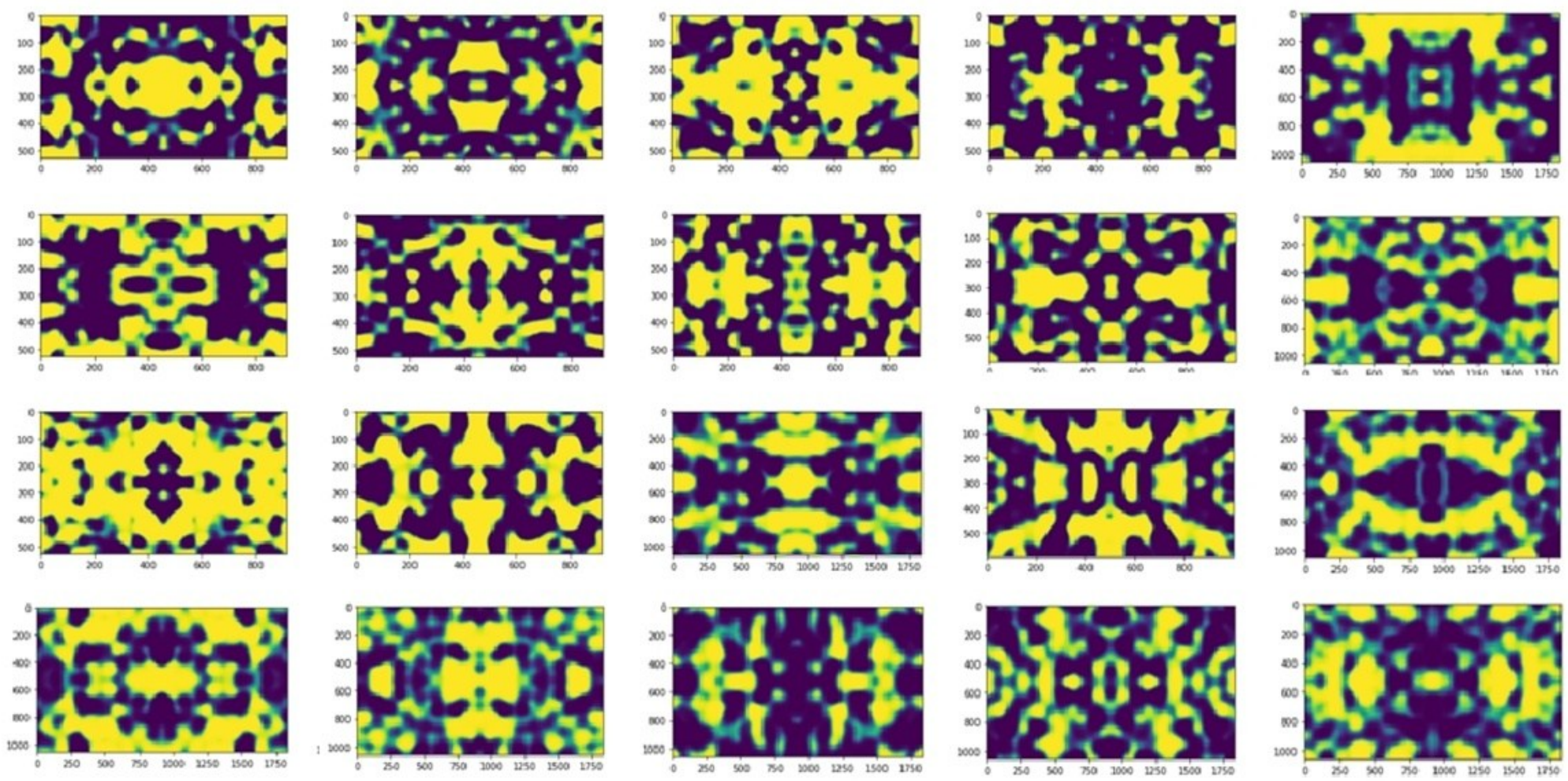

Figure S7. The result without adding Gaussian filtering.

The integration of Gaussian filtering within the PC-GAN framework plays a pivotal role in ensuring the physical realizability and optical performance of generated metagrating designs. By suppressing high-frequency noise and eliminating non-functional nanoscale artifacts, the filtering process enforces fabrication-compatible feature sizes while preserving critical light-manipulating structures. This operation inherently mimics the low-pass spatial frequency response of practical metasurfaces, where subwavelength features below a certain threshold contribute negligibly to phase modulation. The smoothing effect not only enhances edge uniformity but also intrinsically aligns the generated geometries with the non-local electromagnetic response of dielectric nanostructures. As a physics-informed regularizer, Gaussian filtering bridges the gap between neural network outputs and real-world optical constraints, effectively concentrating the design space around solutions that simultaneously satisfy performance targets and manufacturability requirements. This preprocessing step is fundamental to achieving high-yield fabrication success while maintaining the metagratings' ability to control diffraction efficiency, angular response, and chromatic behavior.

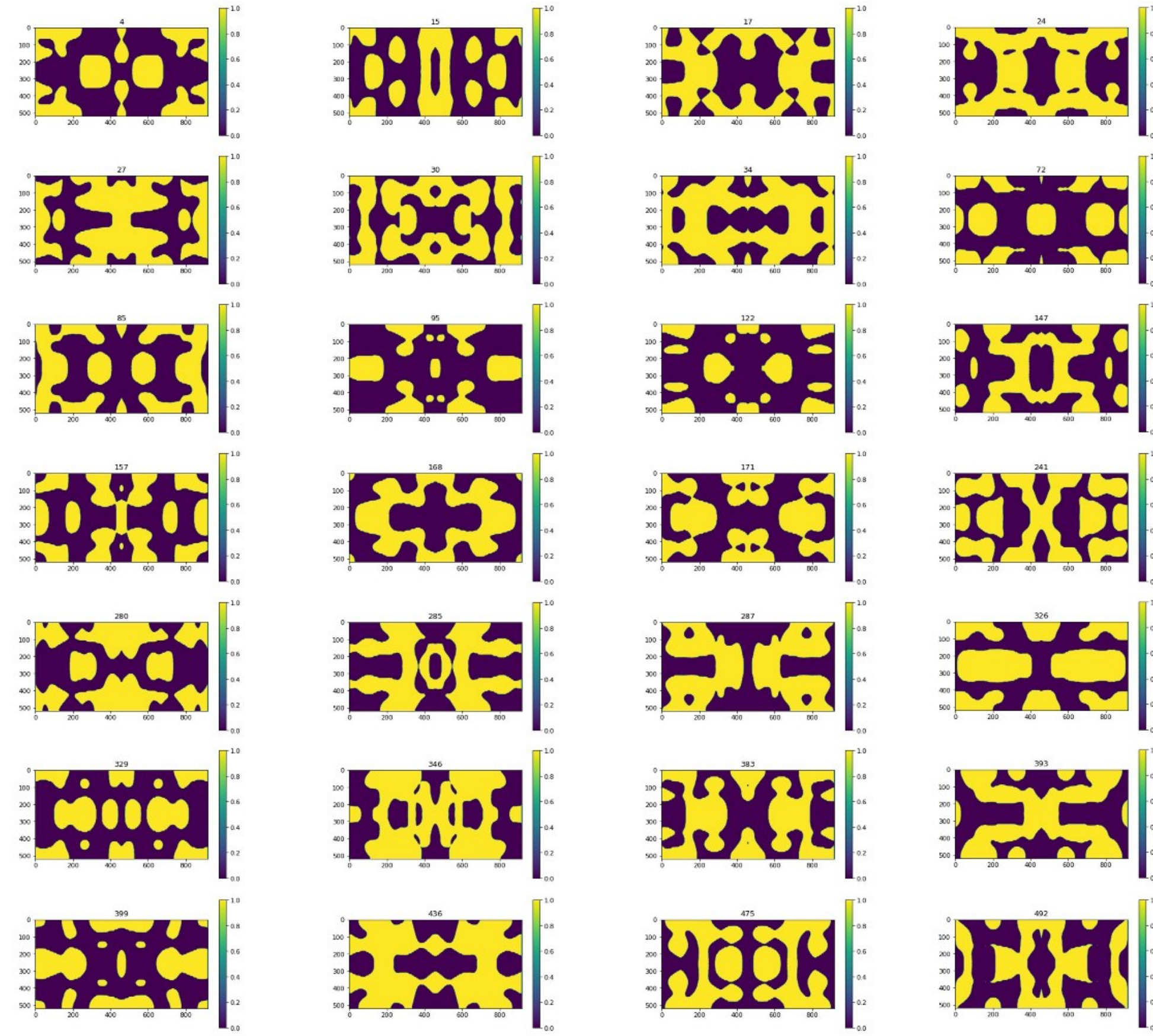

Figure S8. The result without adding DPRR.

The absence of DPRR constraints in PC-GAN-generated metagratings leads to fundamental limitations in optical performance. Without this critical physical constraint, the designs exhibit severe pupil non-uniformity, manifesting as visible fishbone patterns due to uncontrolled energy redistribution during cascaded diffraction. The unconstrained optimization fails to properly manage multi-order interference, resulting in problematic stray light and ghost images that degrade contrast and image fidelity. Furthermore, the angular response becomes unstable, causing significant efficiency variations across the field of view and undesirable color shifts. These deficiencies stem from the generator's inability to automatically maintain the delicate balance between light propagation modes required for effective pupil expansion - a balance that the DPRR metric explicitly enforces through its wavevector matching condition. Ultimately, DPRR-free

designs revert to the same limitations as conventional gratings, losing the key advantages that physics-constrained generative design offers for augmented reality waveguide applications. The constraint serves not just as an optimization target, but as an essential physical principle that maintains the system-level functionality of the metagratings.

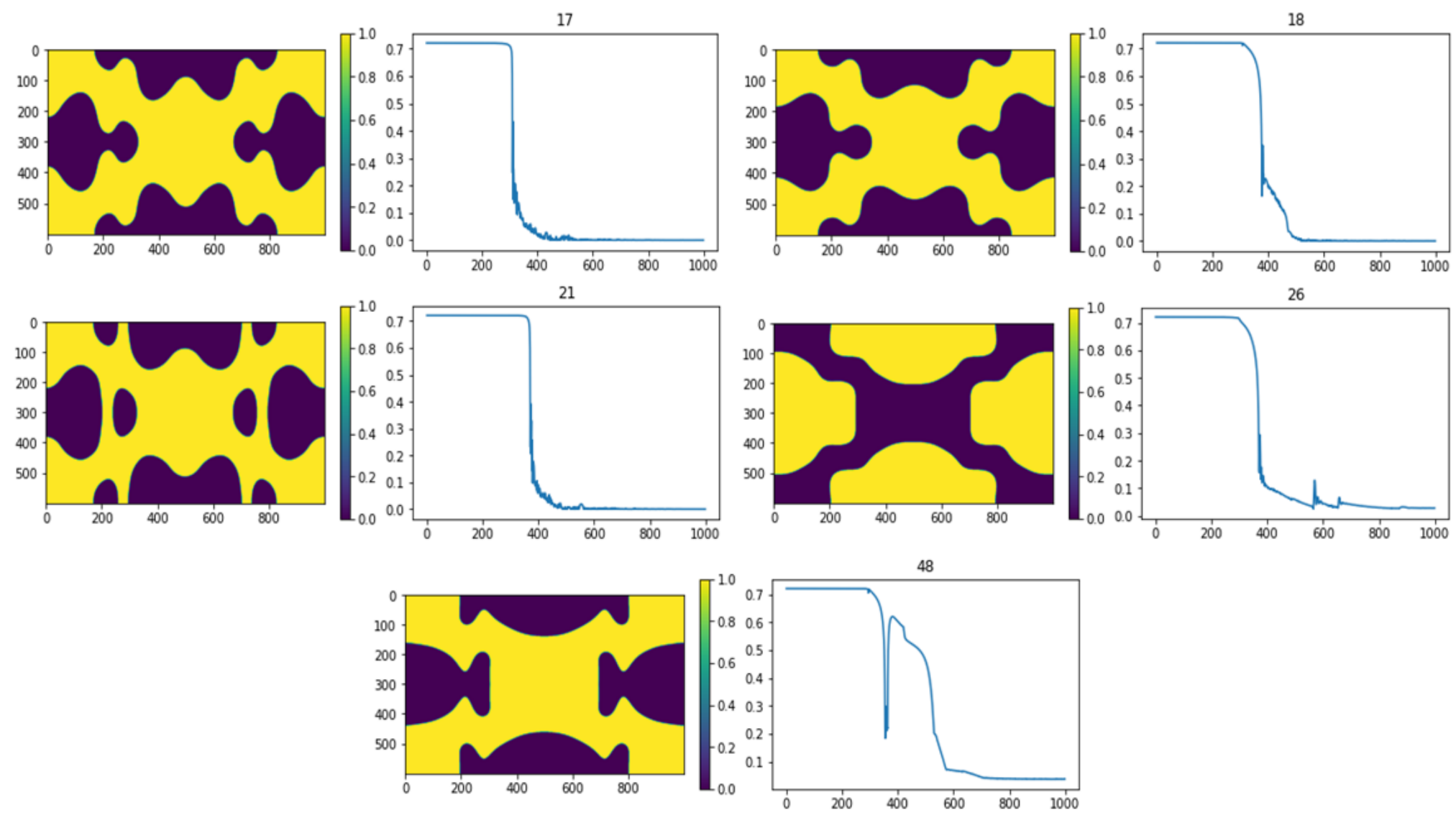

Figure S9. The result adding DPRR.

The consistent appearance of snowflake-like morphologies in DPRR-constrained PC-GAN designs reveal fundamental insights into light-matter interaction optimization. These intricate branching patterns naturally emerge as the generator discovers geometries that simultaneously satisfy three interlinked requirements: balanced energy distribution across diffraction orders, uniform angular response, and efficient phase modulation. The fractal-inspired topology provides multiple length scales for controlling light propagation - the central core regulates primary diffraction efficiency, while the branching sub-structures fine-tune higher-order mode coupling. This hierarchical arrangement enables precise wavefront engineering by creating distributed phase modulation points that collectively maintain the strict DPRR≈1 condition throughout the pupil expansion process. The snowflake geometry's rotational symmetry particularly facilitates uniform

energy transfer in all azimuthal directions, while its self-similar features help suppress wavelength-dependent artifacts through broadband interference effects. These structural characteristics demonstrate how physical constraints can guide generative models toward biologically-inspired photonic architectures that conventional optimization methods might overlook, highlighting the profound connection between constrained adversarial learning and natural photonic design principles.

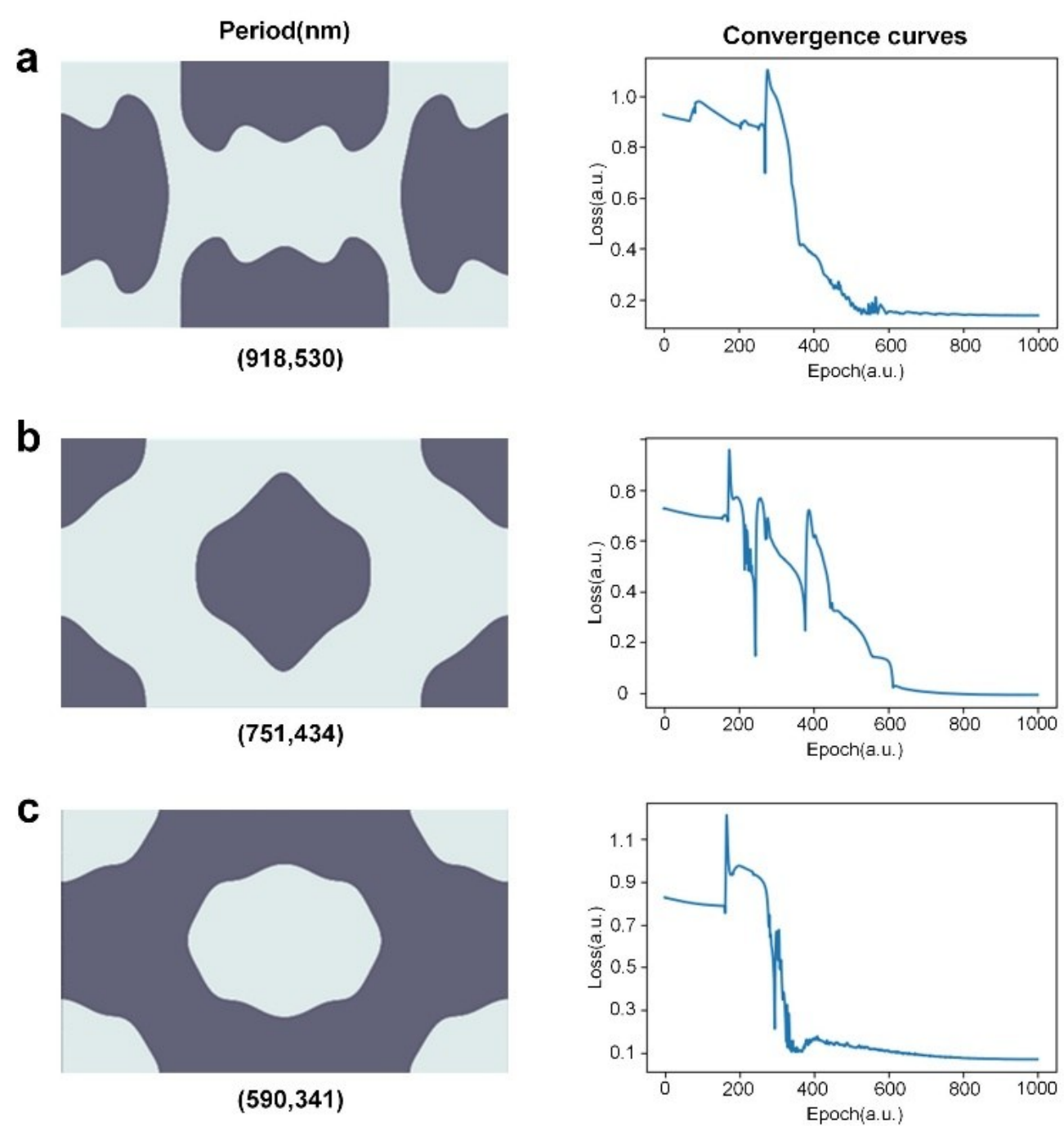

Figure S10. Training convergence and optimized geometries of the PC-GAN for representative grating periods. (a–c) Optimized unit-cell topology (left) and the corresponding loss evolution curve (right) for grating periods of 918 nm, 751 nm, and 590 nm, respectively. The total loss function is composed of the dilated pupil restraint ratio (DPRR) term and the diffraction efficiency terms of the three critical orders (0, 2), (1, 1), and (−1, 1).

The PCT defines the allowable range of grating periods (both horizontal and vertical) through Eqs. (2) and (3) in the main text, ensuring that the ambient-light rainbow effect is completely suppressed across the entire visible spectrum. This acts as a hard boundary condition that fixes the

grating vectors before structural optimization. The DPRR is then embedded into the generator's loss function as a soft constraint, driving the nanoscale geometry toward balanced (0, 2), (1, 1), and (−1, 1) diffraction efficiencies for uniform pupil expansion. Since the PCT locks the period and the grating's azimuthal orientation, the DPRR-guided evolution operates entirely within the design space prescribed by the PCT, leaving no possibility of conflict between the two.

We have included convergence curves (Fig. S10) for representative grating periods (e.g., 590 nm, 751 nm, and 918 nm) at different refractive indices. These curves show the simultaneous evolution of the DPRR and the three critical diffraction efficiencies during both the initial topology optimization (training data generation) and the PC-GAN adversarial training. The overall loss function is composed of the DPRR term and the efficiency terms for the (0, 2), (1, 1), and (−1, 1) orders; as iteration proceeds, this composite loss monotonically decreases and approaches zero. Key observations:

- The DPRR converges toward values above 0.9, with the loss stabilizing within the first 50% of iterations.
- The efficiencies of (0, 2), (1, 1), and (−1, 1) remain within the physically required ranges throughout, and their spectral variance progressively decreases.
- The PCT-dictated period remains strictly fixed, demonstrating that the structural optimization never violates the dispersion constraint.

The PCT narrows the parameter space by fixing the lattice, which dramatically reduces the optimization burden and allows the PC-GAN to focus exclusively on in-cell structural details. This enables the DPRR to converge more rapidly and stably. Conversely, the DPRR ensures that within the PCT-predetermined period, the out-coupling uniformity is optimal, thus making the rainbow-free design practically usable. The two constraints are not competing but rather serve as

successive filters: PCT eliminates chromatic artifacts globally, and DPRR maximizes uniformity locally.

S7. Design parameters and efficiency of SLMG and ELMG

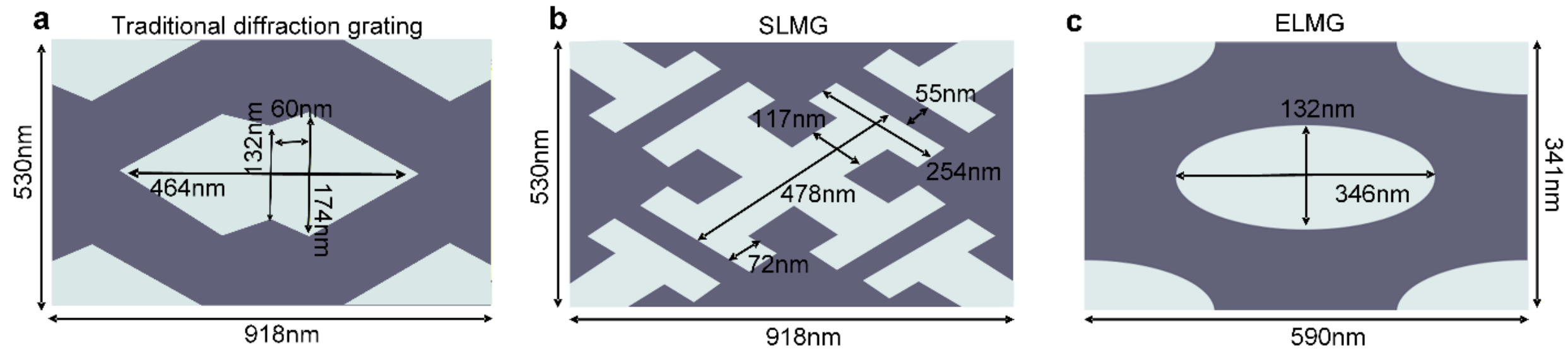

Figure S11. Design parameters of SLMG and ELMG. (a) The traditional diffraction grating has a structure height of 80 nm and a refractive index of 1.7. (b) SLMG has a structure height of 80 nm and a refractive index of 1.7. (c) ELMG employs a height design of 30 nm and a refractive index of approximately 2.6.

During both the generative training and the post-processing topology refinement stages, we enforce practical manufacturing constraints: a minimum feature size of 50 nm, grid snapping to the fabrication resolution, and a Gaussian smoothing layer that eliminates sub-50-nm singularities. These measures ensure that the generated metagrating geometries are not merely theoretical optima but are robust against the resolution limits of electron-beam lithography and nanoimprint lithography.

The ELMG was specifically designed by the PC-GAN as a fabrication-tolerant alternative to the SLMG. The SLMG achieves a higher ideal DPRR (~0.95 in simulation) but relies on freeform features as small as ~50 nm that are challenging for current nanoimprint processes. The ELMG, with its smoother and larger features, exhibits a slightly lower simulated DPRR (~0.8) but retains this performance far more faithfully after replication. Experimentally, the SLMG uniformity drops to 54.3% due to fabrication imperfections, whereas the ELMG uniformity remains close to its design target (49.7% measured). This dual-design strategy explicitly addresses the

sensitivity-performance trade-off and provides a practical pathway for different manufacturing maturity levels.

The PC-GAN was trained over a wide range of refractive indices (n = 1.7, 2.0, 2.6), covering both polymer and SiC platforms. The consistent performance across these material systems confirms that our generated structures are not highly sensitive to moderate refractive index variations caused by deposition conditions or material batch differences. Furthermore, the high-index-contrast SiC platform provides stronger optical confinement, which further reduces sensitivity to dimensional and surface imperfections compared to low-index polymer waveguides.

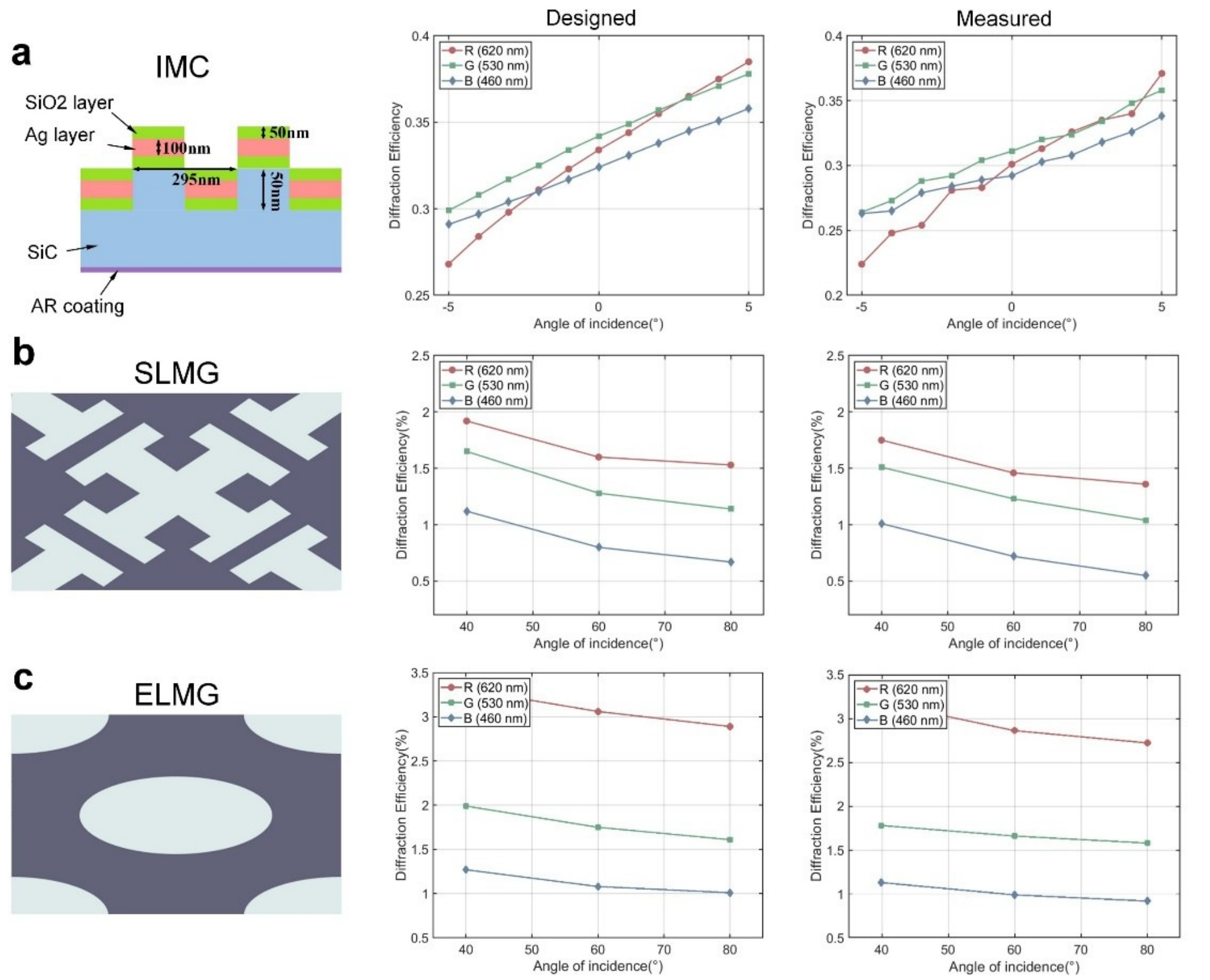

Figure S12. Diffraction efficiency characterization of the fabricated metagrating couplers. (a) IMC. Left: schematic cross-section of the IMC grating design. Middle: simulated coupling-order diffraction efficiency as a function of wavelength for the intended incident angle. Right: corresponding experimentally measured efficiency. (b)Simulated (left) and measured (right) diffraction efficiency of the (0,2) out-coupling order for the SLMG OMC as functions of wavelength and incident angle. (c) Same as (b) but for the ELMG OMC. The SLMG design exhibits a smaller spread in diffraction efficiency across the RGB wavelengths, consistent with its superior color uniformity.

The IMC follows an identical design concept for both the polymer and SiC waveguide platforms, targeting maximum in-coupling efficiency. Figure S12(a) presents the IMC structural schematic, the simulated coupling-order efficiency across the visible band, and the corresponding measured efficiency. The experimental data closely follow the simulated trend, confirming that the fabricated IMC achieves high and broadband in-coupling.

For the OMC, the pupil-expanding orders (0, 0), (1, 1), and (−1, 1) are inherently difficult to isolate accurately in a waveguide geometry because they propagate within the substrate and undergo multiple total internal reflections before detection. We therefore measured the directly out-coupled (0, 2) order, which can be captured with high precision and is the primary channel for image formation toward the eye. Figure S12(b) and (c) show the simulated and measured (0, 2) diffraction efficiency for the SLMG and ELMG OMCs, respectively, as functions of wavelength and angle. The measurements agree well with the design targets, and the SLMG structure exhibits a notably smaller spread among the RGB efficiencies, corroborating its superior color uniformity predicted by the PC-GAN framework.

These new data provide the requested intrinsic validation of the generated metagrating geometries and confirm that the system-level luminance and uniformity gains originate directly from the engineered diffractive behavior.

S8. Schematic diagrams of pupil luminance uniformity of traditional diffraction gratings, SLMG and ELMG

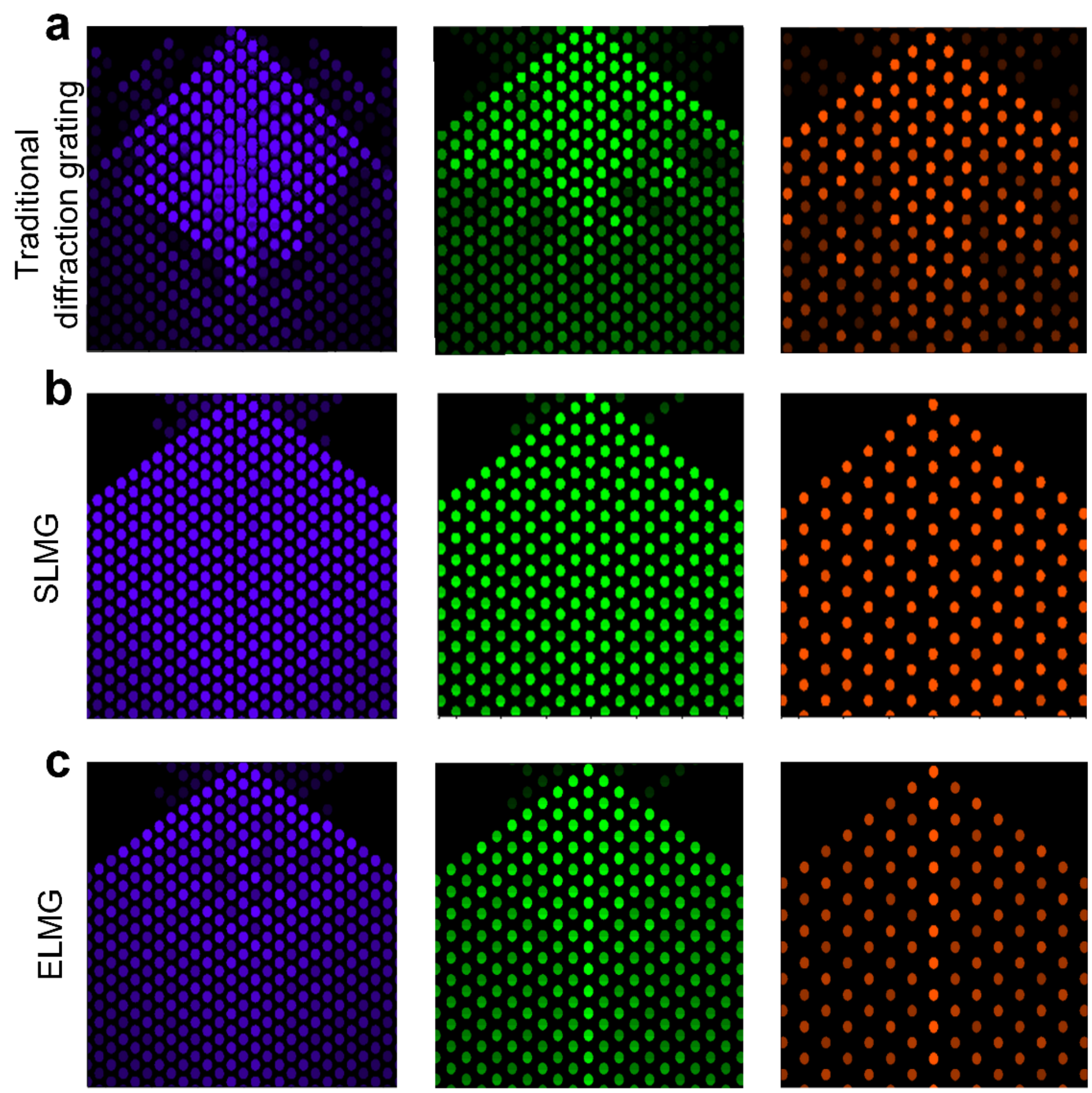

Figure S13. Simulation diagram of pupil luminance uniformity of (a) Traditional diffraction gratings, (b) SLMG and (c) ELMG.

Optical simulations of the waveguide system were conducted using VirtualLab Fusion. Figure S13 compares the simulated pupil luminance distributions among three waveguide architectures: (a) conventional diffractive gratings, (b) SLMG, and (c) ELMG. The conventional design exhibits pronounced fishbone artifacts - characterized by alternating bright and dark bands radiating from the center - with particularly severe manifestation in the blue channel that compromises color fidelity. For direct comparison, both SLMG and ELMG were simulated using identical thick

waveguides with a refractive index of 2.6. The results indicate that both meta-optical structures successfully suppress fishbone patterns via their tailored diffraction properties. The SLMG, however, achieves higher uniformity, as reflected in its near-ideal DPRR. In contrast, the ELMG exhibits slight residual non-uniformity, consistent with its moderately lower DPRR performance. The SLMG completely eliminates artifacts across all RGB channels, confirming its ideal characteristics for full-color augmented reality displays. These simulation results validate that the multi-scale light control capability of the snowflake geometry fundamentally overcomes the limitations of conventional grating designs.

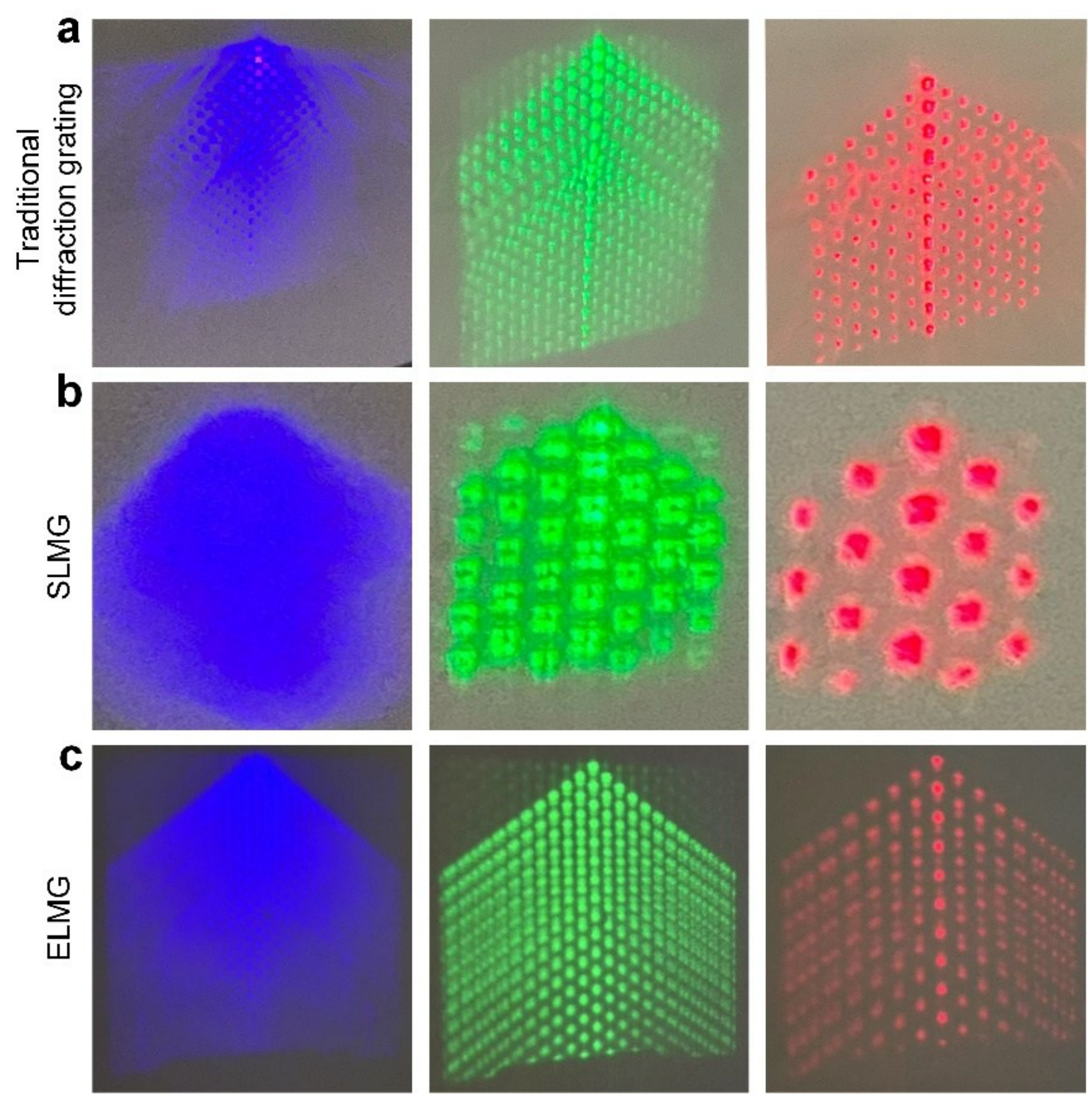

Figure S14. Experimental diagram of pupil luminance uniformity of (a) Traditional diffraction gratings, (b) SLMG and (c) ELMG.

The experimental measurements of pupil luminance uniformity (Fig. S14) show excellent agreement with the simulation results, conclusively validating our design approach. As anticipated, conventional diffractive waveguides exhibit pronounced fishbone patterns with strong center-to-

edge luminance variation, whereas both SLMG and ELMG demonstrate significantly improved performance. It should be noted that the actual processing area of the SLMG is only 1 cm × 1 cm, which limits the expanded pupil sampling area and leads to a relatively smaller eyebox. Nevertheless, the SLMG still exhibits favorable uniformity in luminance, with no obvious fishbone patterns observed. The characteristic alternating bright and dark bands predicted in simulations are accurately reproduced in the experimental results, confirming the fidelity of our physical models. Importantly, the SLMG achieves artifact-free performance across the entire RGB spectrum, with no visible fishbone patterns or chromatic non-uniformity—consistent with its ideal simulated behavior. In comparison, the ELMG exhibits slightly lower yet still substantially enhanced uniformity relative to the SLMG, in line with its simulated DPRR characteristics. To validate this distinction and demonstrate the effectiveness of our designs in mitigating fishbone artifacts, we provide luminance uniformity measurements for all three waveguide implementations: the conventional diffractive waveguide exhibits 18.6% uniformity, while our SLMG and ELMG waveguides achieve 54.3% and 49.7% uniformity, respectively. SLMG represents the ultimate optical solution when advanced patterning becomes available, while ELMG is the optimal choice for manufacturing robustness today.

S9. Comparison of DPRR and MTF distributions of traditional diffraction gratings, SLMG and ELMG

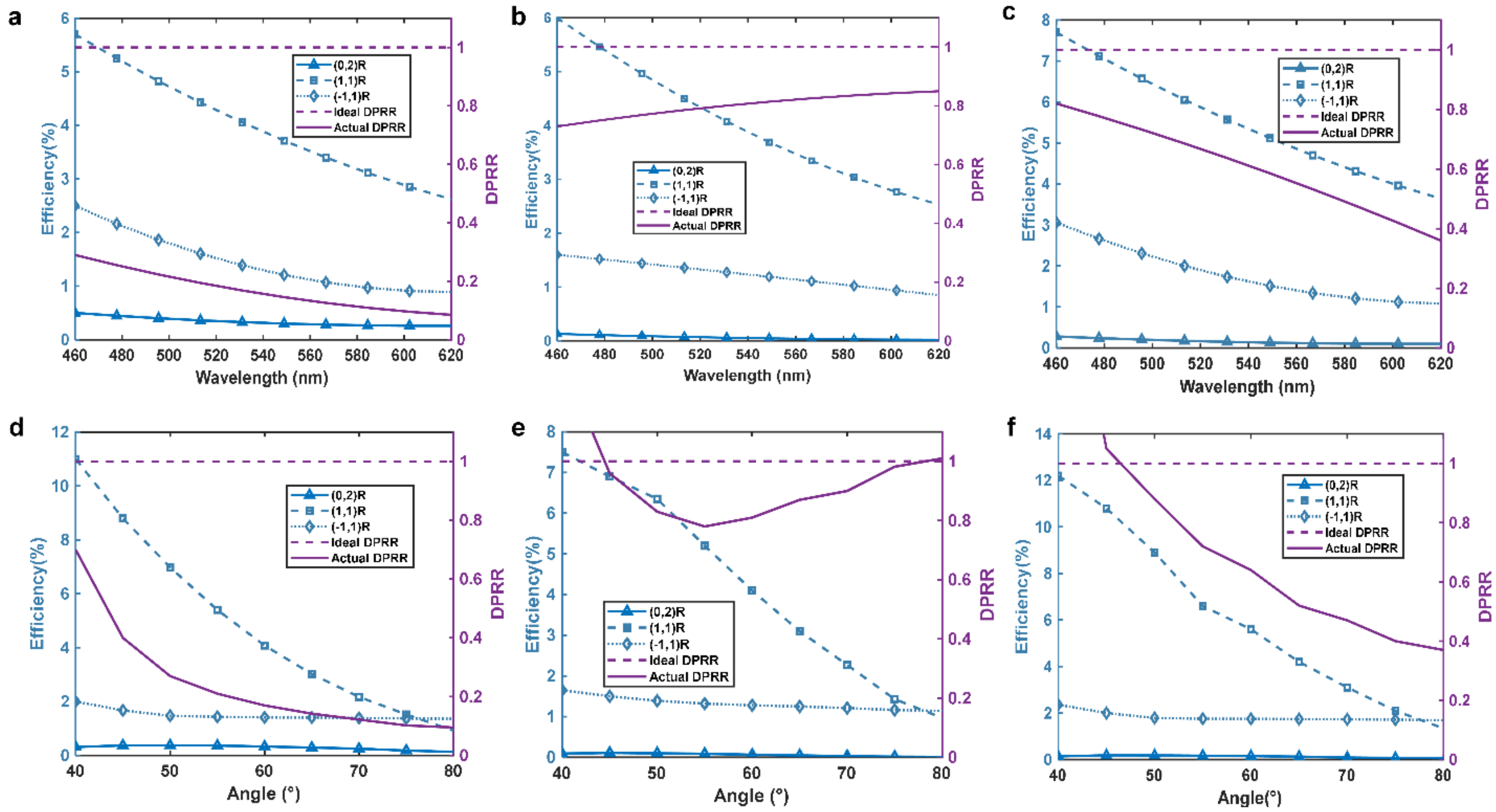

Figure S15. Comparison of DPRR distributions of traditional diffraction gratings, SLMG and ELMG. (a) The wavelength uniformity of traditional diffraction gratings. (b) The wavelength uniformity of SLMG. (c) The wavelength uniformity of ELMG. (d) The angular uniformity of traditional diffraction gratings. (e) The angular uniformity of SLMG. (f) The angular uniformity of ELMG.

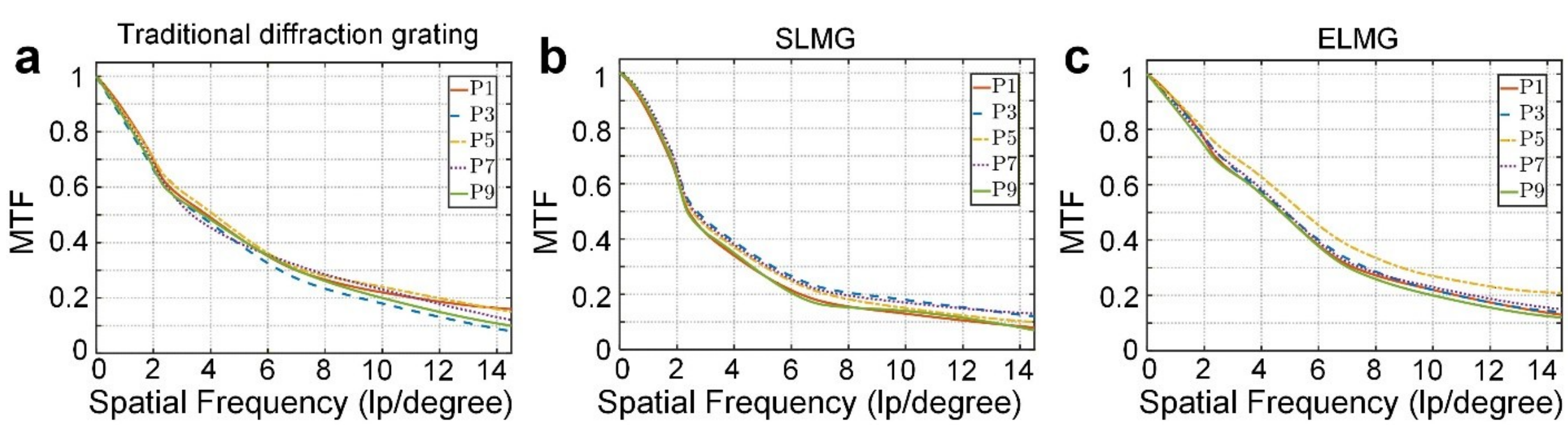

Figure S16. Comparison of MTF distributions of traditional diffraction gratings, SLMG and ELMG.

Note that the SLMG shows slightly lower MTF performance due to its more complex structural design, which leads to higher micro-nano fabrication difficulty and inevitable minor

processing deviations in practice, though the design itself yields a superior luminance uniformity of 54.3%.

S10. Demonstration of the single-layer waveguide display using ELMG

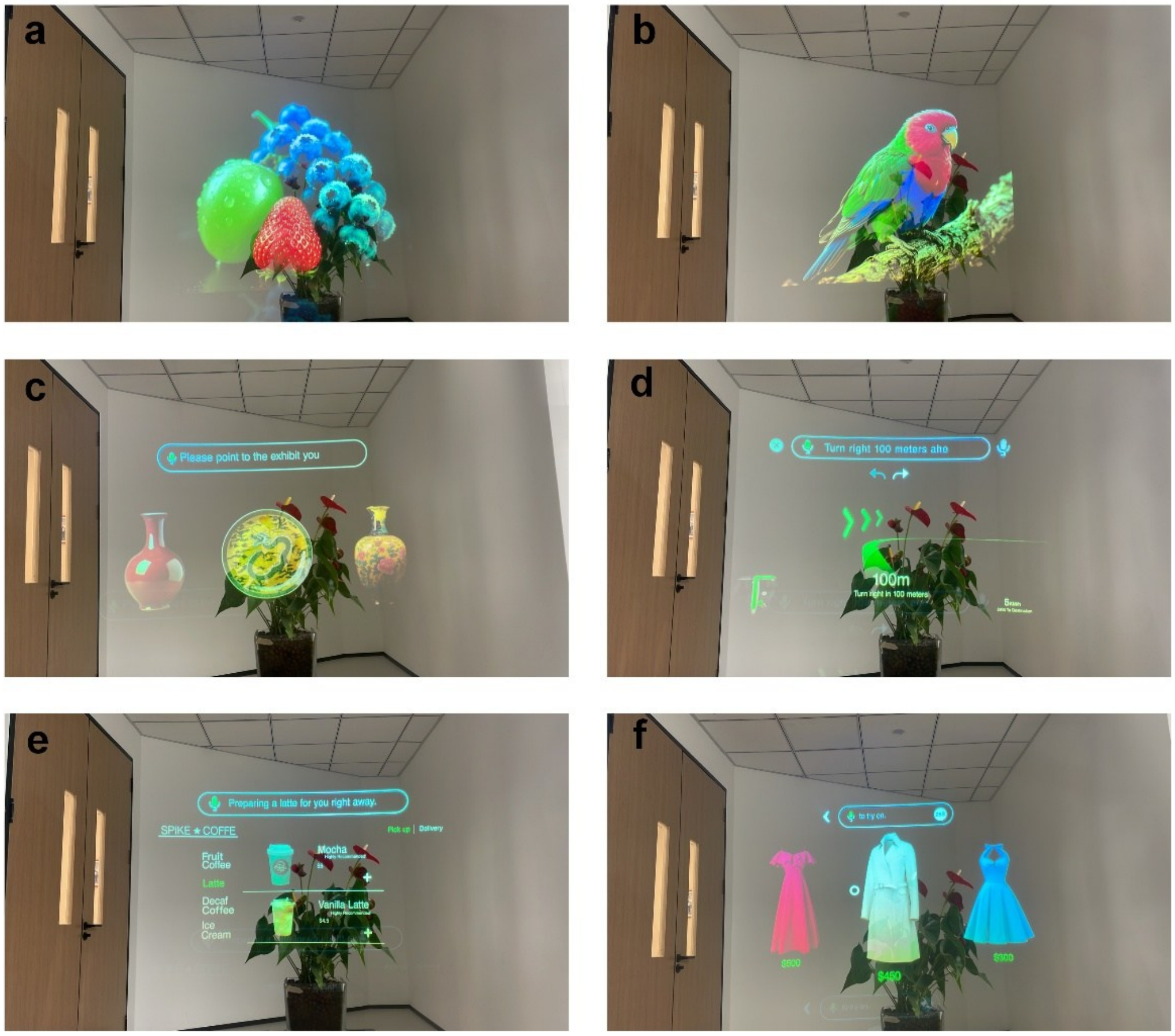

Figure S17. The original experimental image captured by a single-layer waveguide display.

The full-color augmented reality projection tests visually confirm the achieved field-of-view (FOV) capability of our waveguide design, with virtual images seamlessly covering large surfaces such as entire walls.

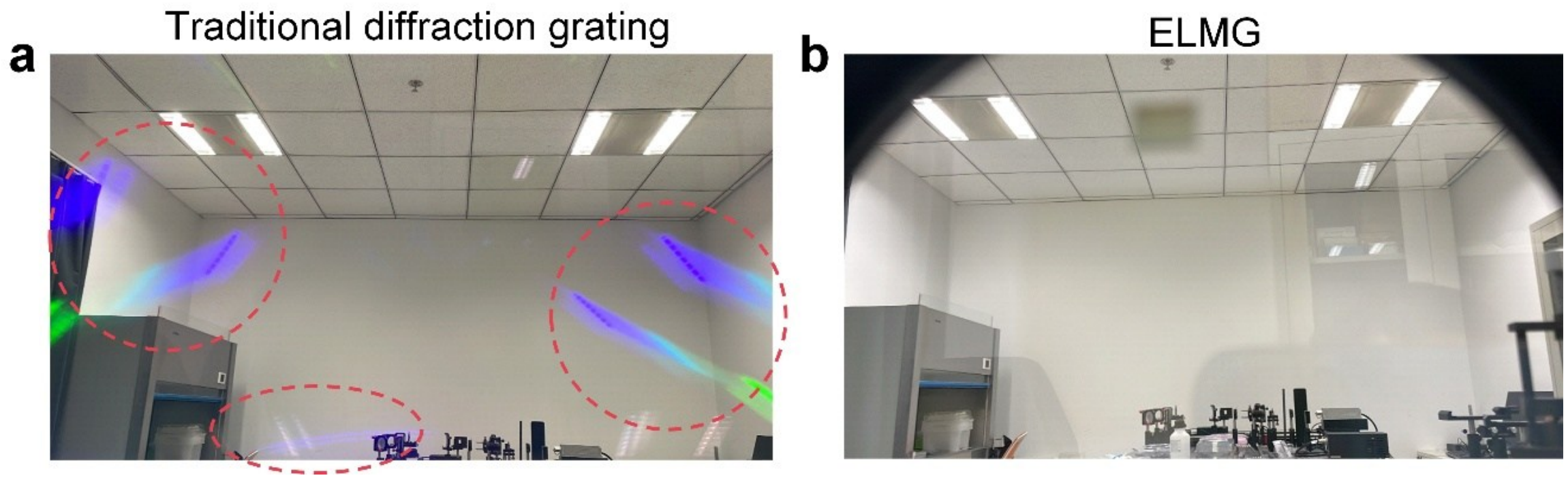

Figure S18. Rainbow fringe test of (a) traditional diffraction grating and (b)ELMG.

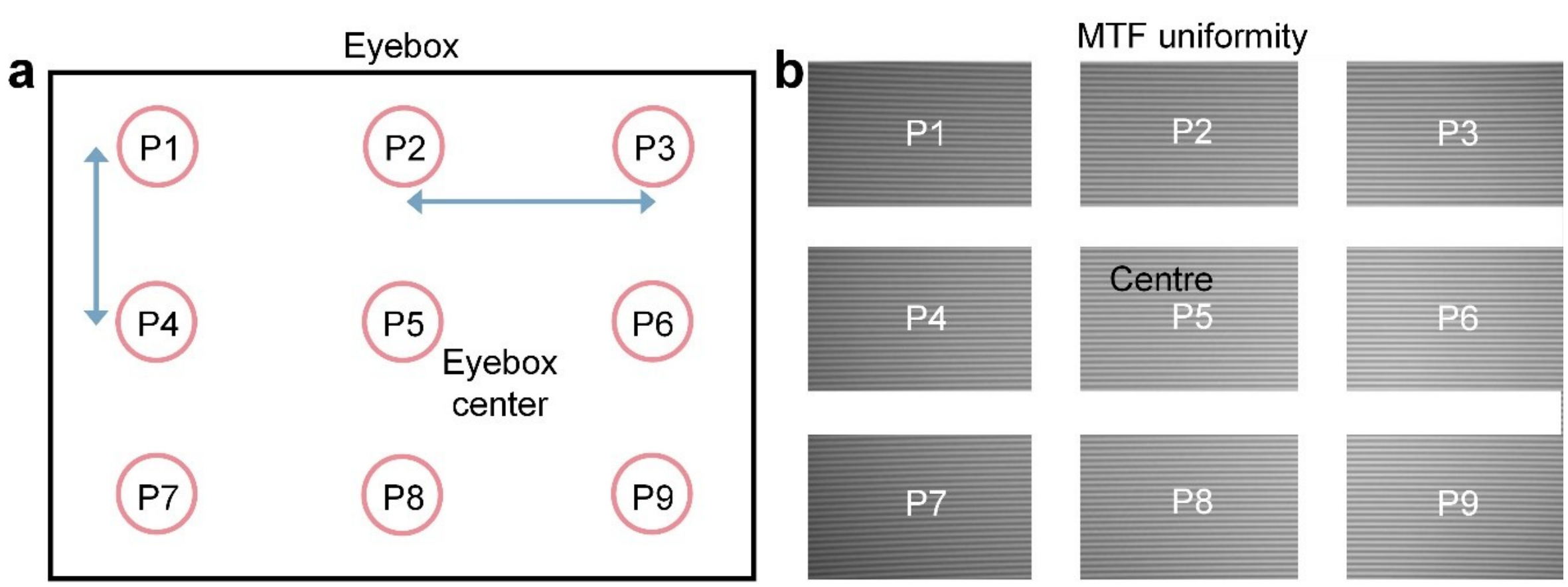

Figure S19. (a) 9-Point Sampling Method. (b)MTF uniformity.

The 9-Point Sampling Method is a standardized optical homogeneity evaluation method. By selecting nine key points in the detection area according to a specific geometric distribution for measurement, the spatial homogeneity of luminance or MTF is systematically quantified. The testing process first demarcates a rectangular area on the exit pupil surface of the light wave, and selects the center point, the midpoints of the four edges and the four corner points as the sampling positions. Subsequently, use a photometer or imaging equipment to measure the luminance values (or MTF values) of each point in sequence, and calculate the maximum deviation rate. This method strikes a balance between detection efficiency and reliability through sparse sampling, and is particularly good at capturing typical defects such as edge attenuation (such as fishbone patterns) and quadrant asymmetry. It is the basic detection scheme for AR/VR optical devices recommended by ISO 9358 and ANSI standards, and is used as a core quality control means in the optical waveguide production lines of enterprises such as Microsoft and Meta.

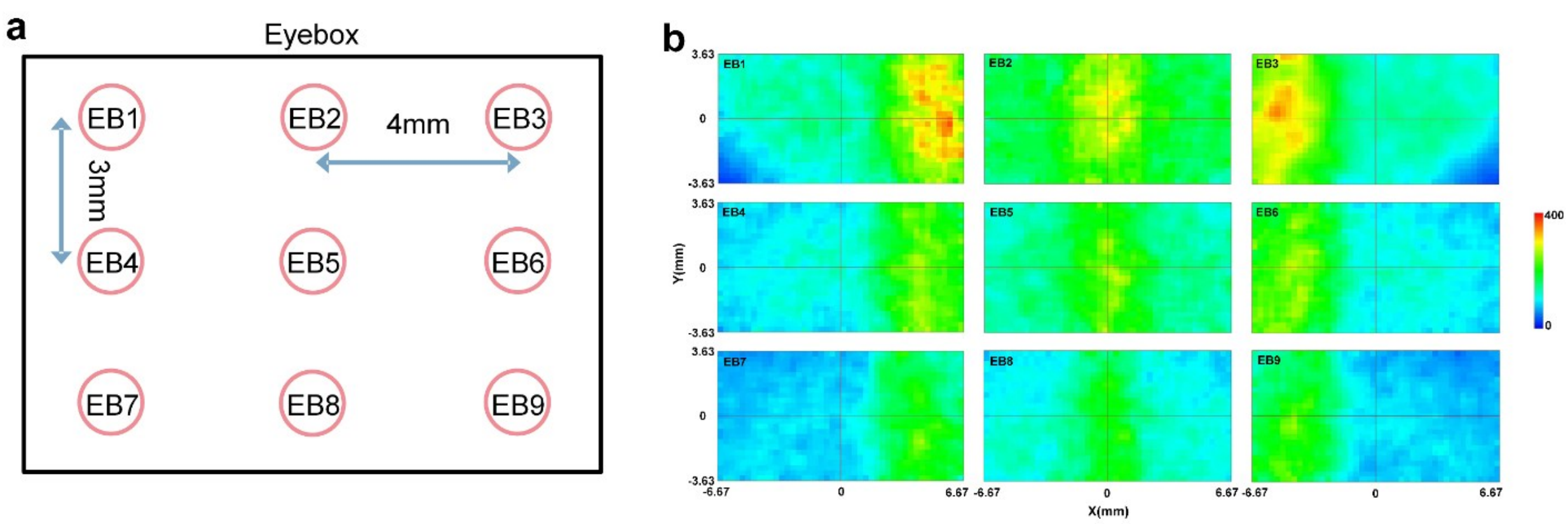

Figure S20. Eyebox characterization. (a) Schematic of the 3 × 3 measurement grid across the eyebox, with horizontal/vertical sampling intervals of 4 mm and 3 mm, respectively. (b) Measured luminance distribution at each eyebox position (EB1–EB9).

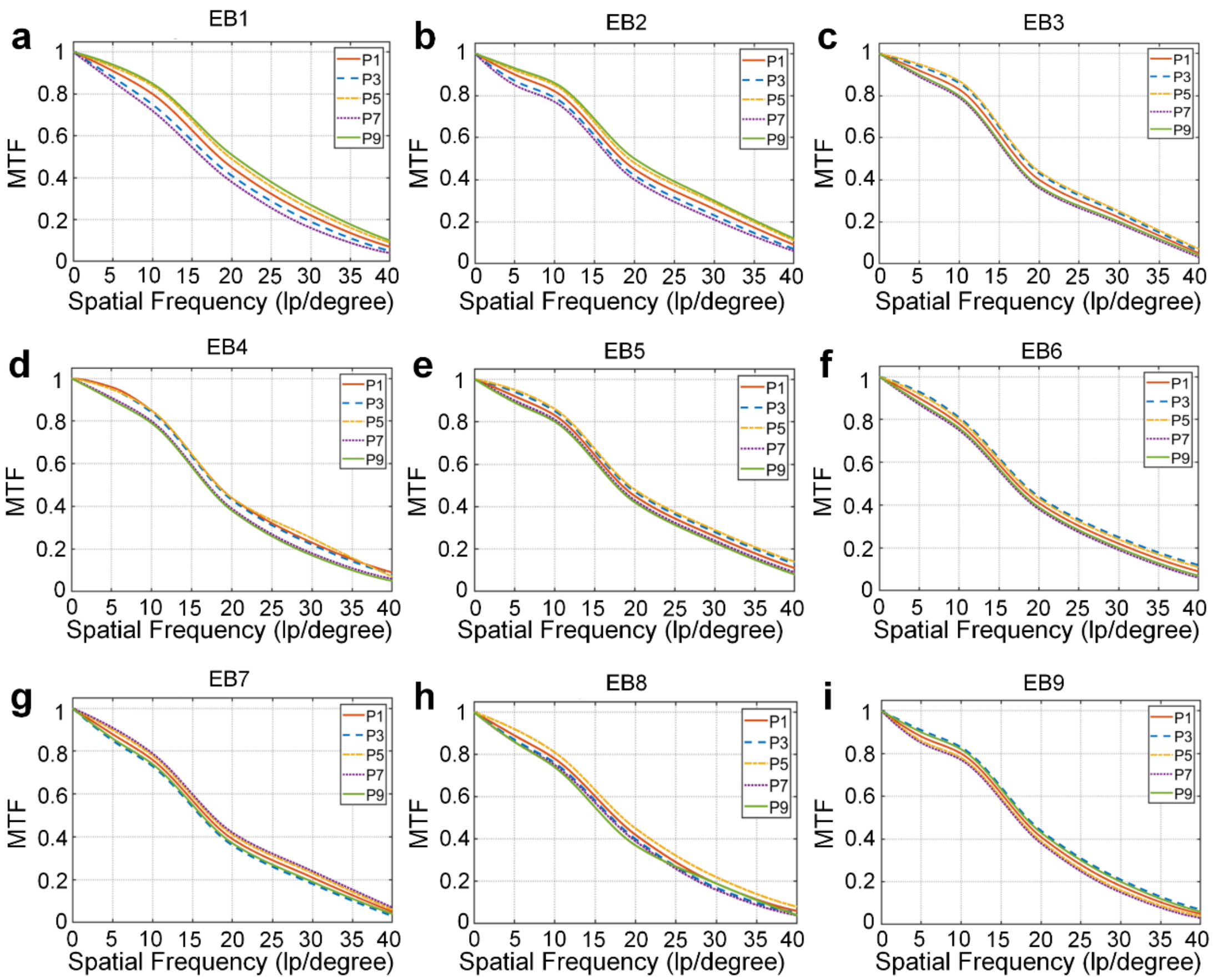

Figure S21. The MTF at nine locations distributed over the eyebox.

Given that 2D pupil expansion inherently produces multiple out coupled beams that fill the eyebox, we assessed the spatial consistency of image quality by measuring the MTF at nine locations distributed over the eyebox (Fig. S21). The standard deviation of MTF at 15 lp/mm across the nine positions is less than 0.05, indicating high uniformity. This result is directly attributable to the DPRR constraint, which balances the efficiencies of the (0, 2) direct out coupling and the (1, 1)/ (−1, 1) secondary expansion paths, ensuring that the point spread function remains stable irrespective of the viewing position within the eyebox.

We quantify the FOV performance and its physical trade-offs. The main challenges in realizing an ultrawide 75° diagonal FOV come from the TIR angular bandwidth of the waveguide substrate and the angular spectral response of the metagrating. Full-angle simulations across the

entire 75° FOV confirm excellent angular uniformity. As a physically expected trade-off for such an ultrawide FOV, the overall luminance efficacy decreases to 76 nit/lm (Fig. S22).

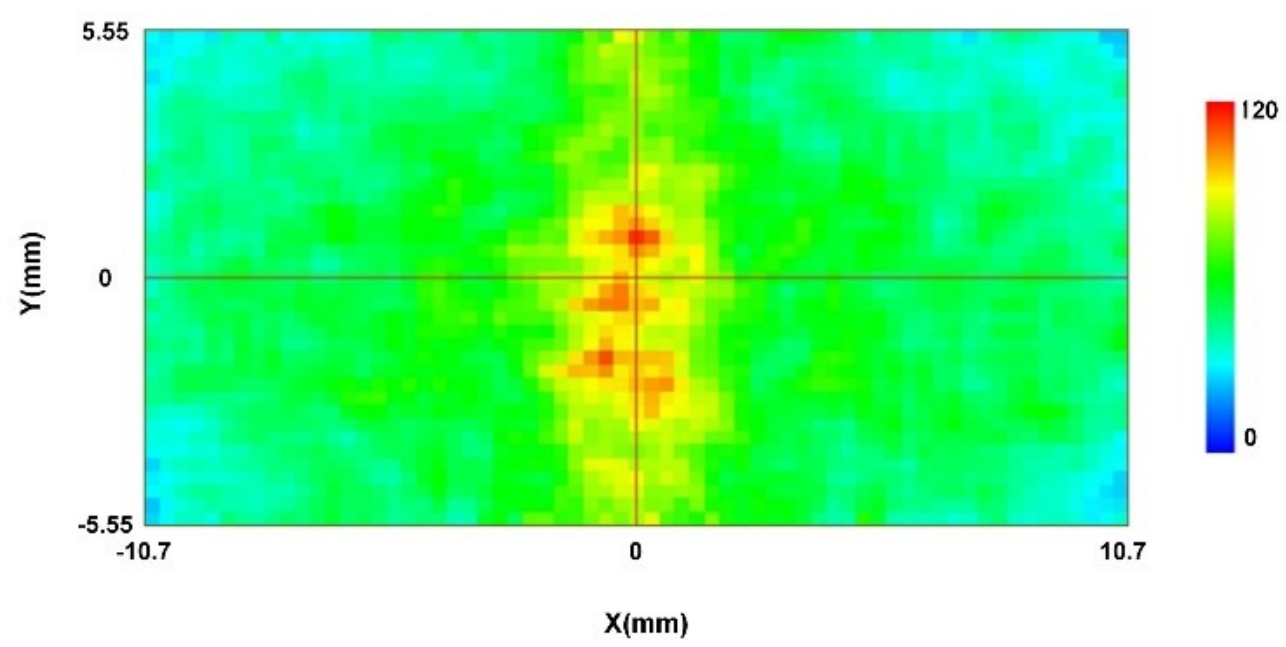

Figure S22. 75° central eyebox luminance uniformity

We then provide a complete quantitative characterization of the eyebox. The eyebox is located 20 mm in front of the out-coupling region, as shown in the corresponding Fig. 2i. Given the optical engine FOV (75° diagonal, 16:9 aspect ratio), the maximum horizontal and vertical half-angles are $\max(\theta_H) = 33.7°$ and $\max(\theta_V) = 20.6°$, respectively. Combined with the output coupling grating dimensions ($L = 33.8$ mm, $W = 28.6$ mm) and the 20 mm eye relief, the geometric eyebox that supports full FOV reception is calculated to be 7.1 mm × 13.5 mm. Considering luminance uniformity—especially non-uniformity in the vertical direction—and validated by both simulation and measurement, the effective eyebox that meets display quality requirements is determined to be 7.1 mm × 7.0 mm.

In conclusion, the achieved performance—combining a 75° diagonal FOV and a 7.1 × 7.0 mm effective eyebox—demonstrates that the proposed PC-GAN design successfully overcomes the conventional étendue-limited trade-off between FOV and eyebox, enabled by the 2D pupil-expansion architecture.

Color uniformity

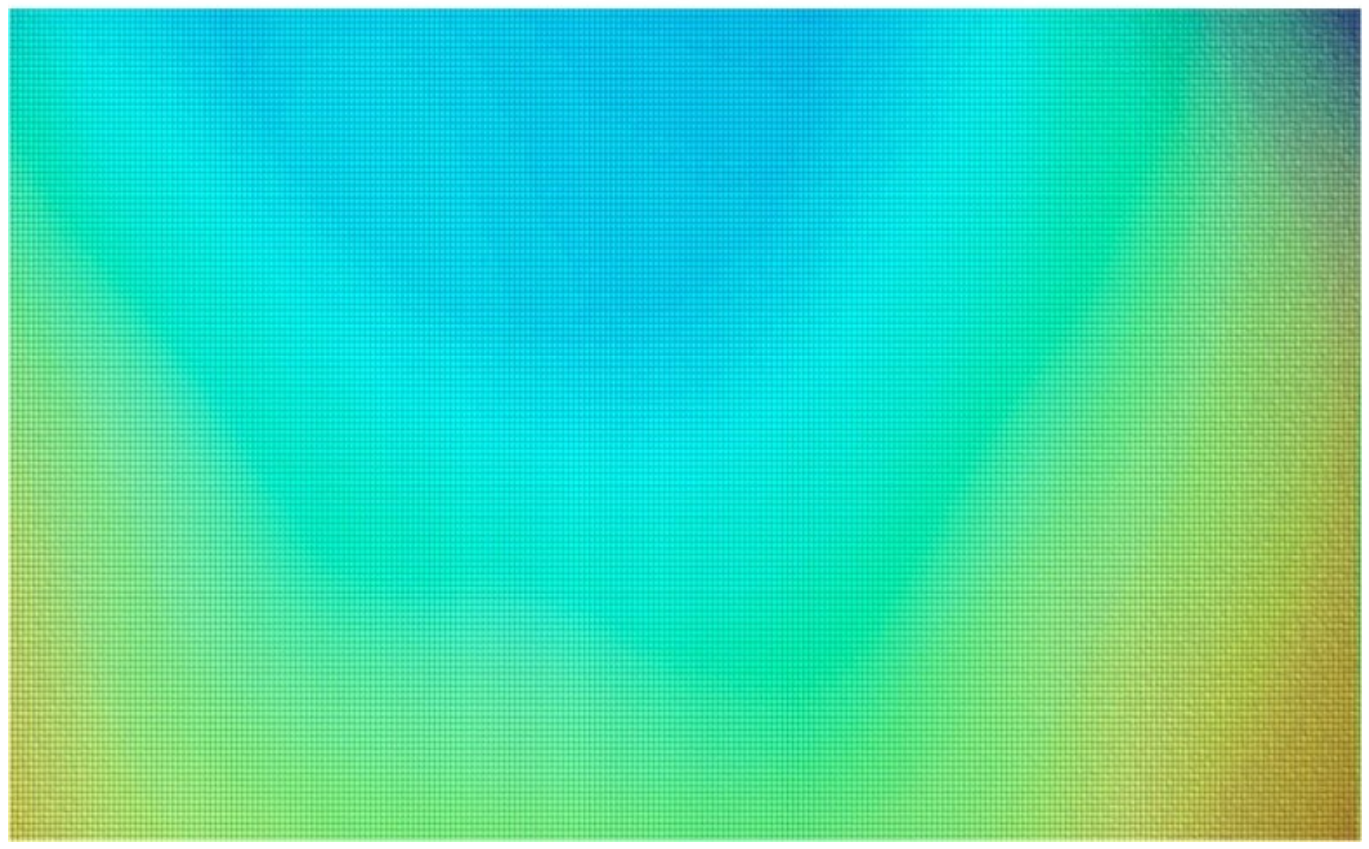

Figure S23. Color uniformity analysis (color uniformity of 0.102)

We have now reported the complete efficiency budget. The optical engine employs a DLP projector (Coretronic, 1280 × 720 resolution) with an output luminous flux of 6 lm. The measured luminance efficacy averaged across the field of view is approximately 180 nit/lm. The efficiency of optical power coupled into the waveguide via the IMC is 19%, and the overall system efficiency from in-coupling to out-coupling is 2.03%. This low per-interaction OMC efficiency is a necessary physical constraint imposed by the large FOV and enlarged outcoupling area. A higher efficiency would deplete the guided beam prematurely and lead to severe non-uniformity across the expanded pupil.

A custom stray light characterization platform was built to simulate ambient light incident on the waveguide output coupler under daily usage conditions. As shown in Supplementary Fig. S24a, the angular test configuration consisted of the waveguide sample rotating from 10° to 170° in 20° steps. The experimental setup is shown in Supplementary Fig. S24b. A luminance colorimeter was aligned with the central OC region and positioned at the eye relief distance, simulating the human eye FOV. The single-FOV stray light generation ratio was 0.03%—two orders of magnitude below typical diffractive waveguide stray light levels—confirming that the PCT-optimized grating period effectively suppresses wavelength-dependent diffraction of ambient light.

The visual evidence further corroborates the quantitative results. Supplementary Fig. S24c and S24d show the ELMG waveguide placed horizontally and vertically, respectively, with no observable rainbow coloration in either orientation. In striking contrast, Supplementary Fig. S24e and S24f show a conventional diffractive waveguide under identical conditions, where pronounced rainbow artifacts are clearly visible across the field of view regardless of waveguide orientation. This combination of a 0.03% quantitative stray light ratio and direct side-by-side imaging comparison directly substantiates the "rainbow-free" claim and demonstrates the practical effectiveness of the PCT-based dispersion-engineered design.

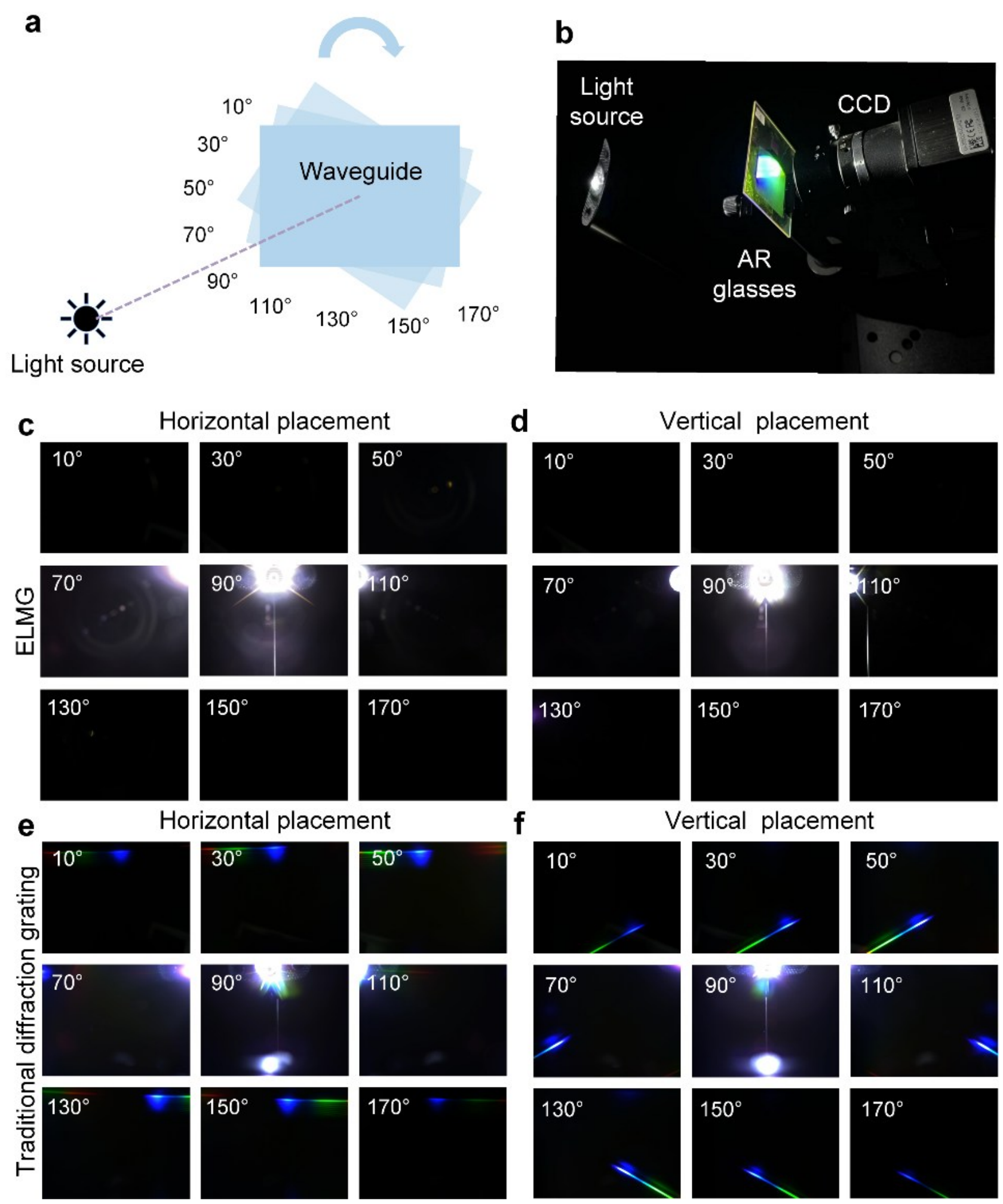

Figure S24. Quantitative stray light characterization for rainbow artifact suppression. (a) Schematic of the angular test configuration, showing the nine sample rotation angles (10°–170° in 20° steps). (b) Photograph of the experimental setup. (c, d) Images captured through the ELMG waveguide placed horizontally (c) and vertically (d), showing complete absence of rainbow artifacts. (e, f) Images captured through a conventional diffractive waveguide placed horizontally (e) and vertically (f), showing pronounced rainbow artifacts across the field of view.

S11. Comparison between traditional design and PC-GAN

Table S1. Comparison between traditional design and PC-GAN.

Dimension	Traditional design	PC-GAN
Wavelength range	Single wavelength	Visible light (400-700nm)
Maximum FOV	30° ~35°	>50°
Efficiency	Only one diffraction order can be optimized	Collaborative optimization of multiple physical quantities
Uniformity	The fishbone artifacts	No fishbone artifacts
Dispersion control	The rainbow effect	No rainbow effect
Design method	Wavelength and Angle segmented optimization	End-to-end joint generation
Process complexity	Multi-layer stacking (2 to 3 layers)	Single-layer integration
Structure	Rhombus, cylinder	Snowflake-like
Design speed	Days or even months	A few minutes

The PC-GAN framework demonstrates significant advantages over traditional design approaches across multiple critical dimensions. Unlike conventional methods limited to single-wavelength optimization and narrow field-of-view (30°–35°), PC-GAN enables full visible spectrum operation (400–700 nm) with extended FOV exceeding 50°. While traditional designs struggle with efficiency trade-offs between diffraction orders and require multi-layer stacking, PC-GAN simultaneously optimizes multiple diffraction orders in a single-layer architecture. This advanced approach eliminates two persistent artifacts of diffractive waveguides: fishbone patterns (through uniform energy redistribution) and rainbow effect (via dispersion control). The snowflake-like geometries generated by PC-GAN contrast sharply with basic rhombic or cylindrical structures of conventional gratings, reflecting their inherent capability for complex light manipulation. Furthermore, PC-GAN's end-to-end joint generation paradigm replaces the

traditional segmented optimization process, reducing design time from months to minutes while maintaining fabrication compatibility. These improvements collectively establish PC-GAN as a transformative methodology for high-performance metagratings design.

Table S2. Comparison of Key Performance Indicators for Full-color Display Diffraction Waveguides.

Feature	Meta Orion (meta)	3D holographic augmented-reality (23)	Single-layer waveguide (18)	Achromatic metagrating (17)	This work
Release Date	2024.09.26	2024.05.08	2025.02.25	2025.04.30	-
Pupil Expansion Mode	Unknown	One-dimensional	One-dimensional	One-dimensional	Two-dimensional
Grating Type	Unknown	1D	1D	1D	2D
Waveguide Material	SiC	SiN	SiN	SiN	Polymer and SiC
Field of View (FOV)	70°	11.7°	45°	20°	75°
Luminance Efficiency (RGB display)	Unknown	Unknown	Unknown	Unknown	180 nit/lm
Fishbone Artifacts Elimination	Unknown	No	No	No	Yes
Rainbow Artifacts Elimination	Unknown	No	No	No	Yes
Brightness Uniformity (RGB display)	Unknown	R61.7%, G91.2%, B98.3% (Typical 58.9%, 47.7% and 88.8%)	R72.8%, G85.1%, B46.3%	15.23%	49.7% (Typical below 20%)
IMC Efficiency	Unknown	About 5%	5%	24%	30%

OMC Efficiency	Unknown	About 2%	2%	1%-24%	1.5% (Typical about 1.5%)
Technical Phase	Demo	Demo	Demo	Demo	Mass production

Among all compared systems, our work achieves the widest FOV (75°), significantly exceeding the 1D pupil-expanding waveguides (11.7°–45°). This is a direct consequence of our 2D pupil expansion architecture, which simultaneously replicates the beam along both horizontal and vertical axes within a single waveguide layer, decoupling the FOV from the micro display. To the best of our knowledge, our work is the only single-layer waveguide that explicitly and quantitatively demonstrates simultaneous elimination of both fishbone artifacts and rainbow effects.

It is important to emphasize that most of the compared works (Refs. 17, 18, 46) employ one-dimensional (1D) pupil expansion, for which luminance uniformity is a considerably less stringent problem. Even so, the best 1D systems exhibit significant channel-to-channel variation (e.g., Ref. 18: R 72.8%, G 85.1%, B 46.3%) and the reported values typically reflect single-channel peak-to-valley rather than a simultaneous white-light measurement. Two-dimensional (2D) pupil expansion, which is required for compact, large-eyebox architectures, introduces cascaded out-coupling interactions that inherently amplify non-uniformity. As a result, the white-light uniformity of conventional 2D diffractive waveguides is notoriously poor—typically below 20% and accompanied by visible fishbone artifacts. Our PC-GAN-optimized SLMG and ELMG waveguides achieve 54.3% and 49.7% white-light uniformity, respectively, in a full 2D expansion geometry. This represents a 2.5× to 3× improvement over the state-of-the-art for 2D waveguides. The key enabler is the DPRR constraint, which explicitly balances the direct and secondary out-coupling paths that govern the 2D pupil replication process.

Our measured IMC coupling efficiency of 30% is the highest among all compared systems operating at wide FOVs. Our OMC single-interaction efficiency (~1.5%) is consistent with the typical level for 2D pupil-expanding waveguides and is a necessary physical consequence of the multi-interaction out-coupling required for uniform 2D expansion. Unlike 1D systems (where a single out-coupling event can achieve 1%–24%), a 2D system must distribute the guided optical power over many replicated exit pupils, inherently reducing the per-interaction efficiency. Despite this, our system achieves a luminance efficacy of 180 nit/lm and a display brightness of 1100 nits, validating that the efficiency is sufficient for practical AR applications. Notably, our work is the only entry in the comparison table that reports luminance efficacy (180 nit/lm), a figure of merit that directly links the optical throughput to the display brightness.

In summary, the quantitative comparison demonstrates that our PC-GAN-based single-layer waveguide uniquely combines an ultrawide 75° FOV, complete fishbone and rainbow artifact suppression, and the highest in-coupling efficiency among wide-FOV systems, while maintaining uniformity 2.5–3× higher than conventional 2D designs. These results substantiate the competitive edge of the proposed framework and its practical significance for next-generation AR displays.

S12. Fabrication of the single-layer waveguide display using SLMG

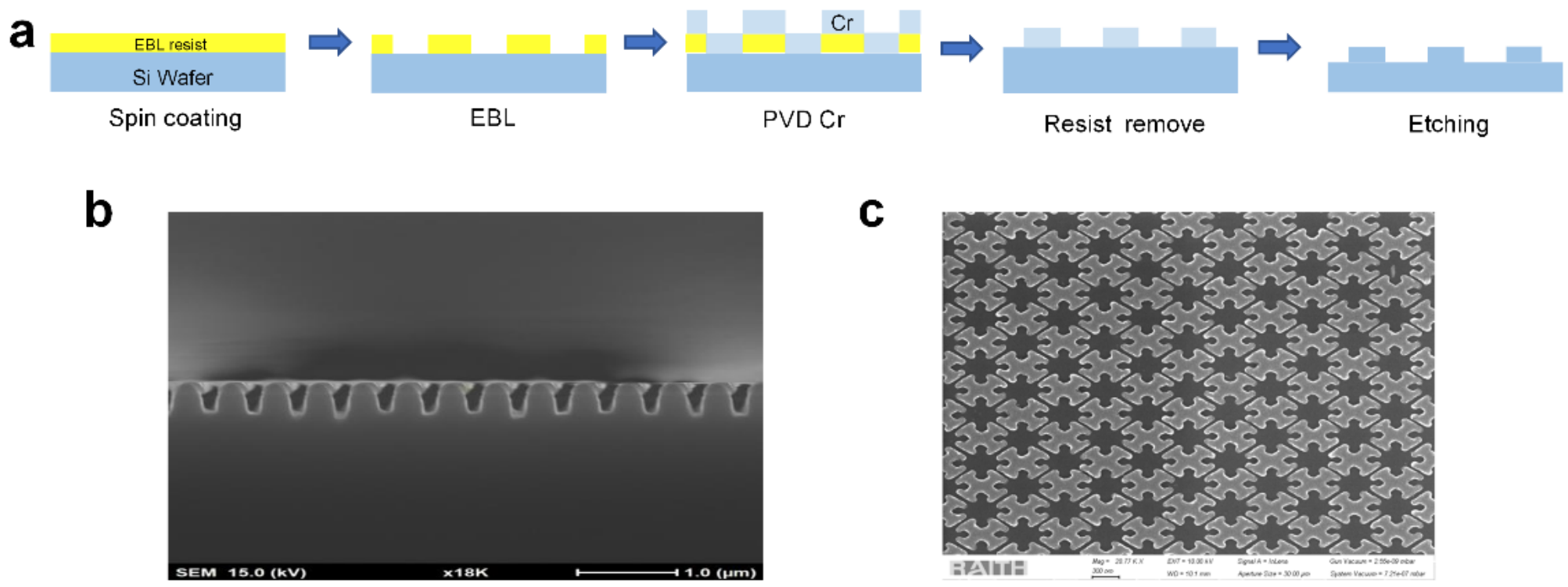

Figure S25. (a) Master processing process. (b) The scanning electron microscope images of IMC. (c) The scanning electron microscope images of OMC.

The fabrication of the SLMG started from the spin coating of the resist (PMMA 950 A5) on a Si substrate, where 300 nm resist was obtained by a spin speed of 4000 rpm. Electron beam lithography (Raith EBPG 5200) was then introduced to write the pattern under the write voltage of 100 kV with a dose of 750 μC/cm^2. Subsequently, the process of the PMMA was executed by immersing the sample into the MIBK: IPA=1:3 solution for 40 s, followed by 100 nm Cr deposition by the E-beam deposition. Then, the sample was immersed for 20 h for the lift-off, and the snowflake-like pattern of Cr was taken shape as the mask. An inductively coupled plasma reactive ion etching (ICP) was utilized to etch the Si to the target depth. Finally, the Si nanostructures were realized after removing the residual Cr by remover of ammonium cerium nitrate and perchloric acid solution.

S13. Fabrication of the single-layer waveguide display using ELMG

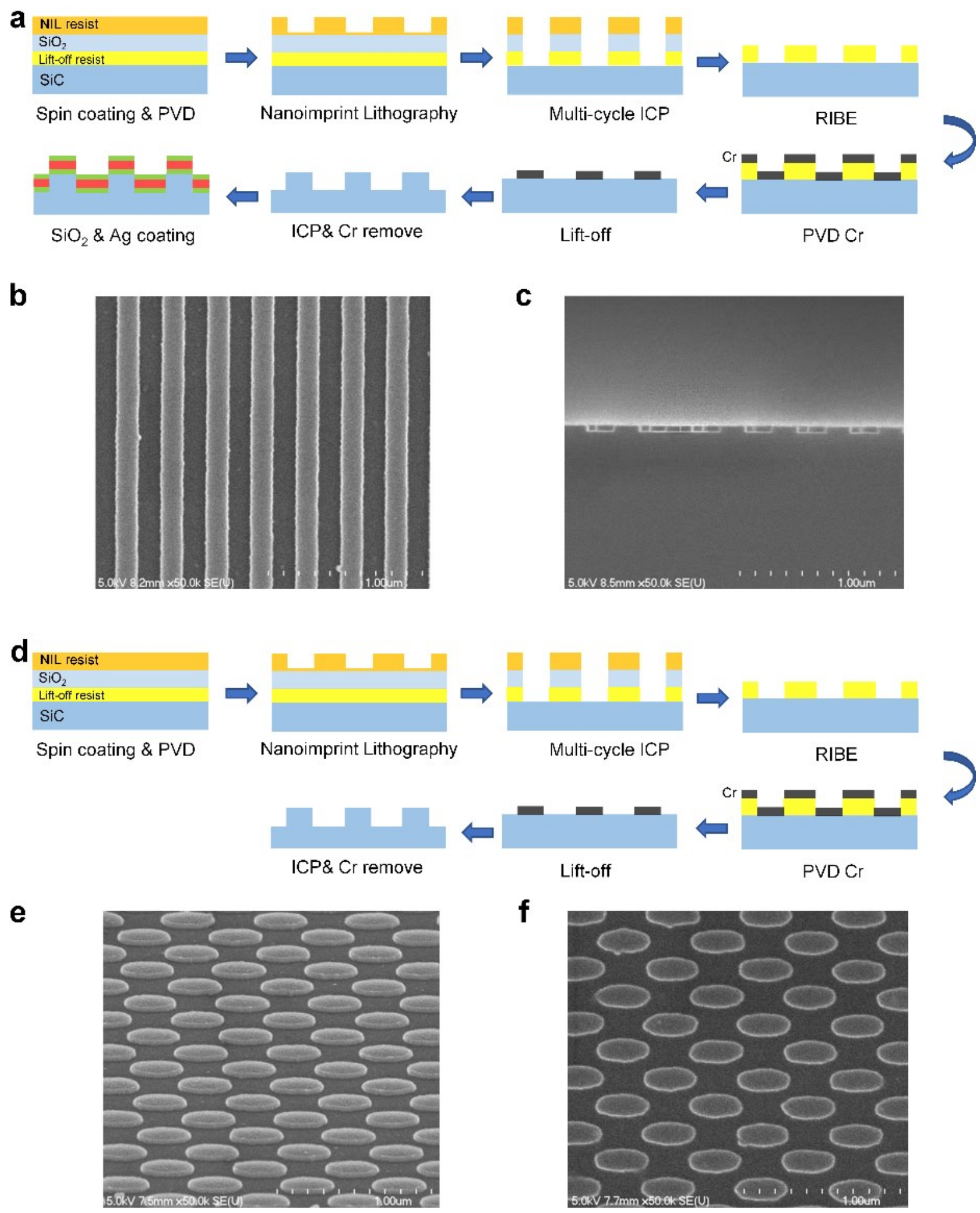

Figure S26. (a) Master processing process of IMC. (b) and (c) The scanning electron microscope images of IMC. (d) Master processing process of OMC. (e) and (f) The scanning electron microscope images of OMC.

Our SiC waveguide AR glasses integrate a high-refractive-index SiC waveguide, fabricated through an optimized nanoimprint lithography (NIL) process (Fig. S26). Unlike conventional diffractive waveguides that rely on soft resist materials prone to damage, we used a robust NIL-to-lift-off process to enhance durability and optical performance. This method replaced traditional NIL-to-etch techniques, which suffer from rapid resist degradation during etching, with a metal hard mask (Cr) that improves etch selectivity and minimizes defects. The process began with high-

precision electron beam lithography (EBL) on a silicon master (Raith EBPG, 100 kV, 10 nA exposure), followed by inductively coupled plasma (ICP) etching to achieve deep, high-fidelity grating structures. To extend mold longevity, the pattern was transferred to a PET film via UV-curing and anti-adhesion treatment, enabling repeated imprinting without degradation.
